# Off-Target Analysis in Gene Editing and Applications for Clinical Translation of CRISPR/Cas9 in HIV-1 Therapy

**DOI:** 10.3389/fgeed.2021.673022

**Published:** 2021-08-17

**Authors:** Andrew Atkins, Cheng-Han Chung, Alexander G. Allen, Will Dampier, Theodore E. Gurrola, Ilker K. Sariyer, Michael R. Nonnemacher, Brian Wigdahl

**Affiliations:** ^1^Department of Microbiology and Immunology, Drexel University College of Medicine, Philadelphia, PA, United States; ^2^Center for Molecular Virology and Translational Neuroscience, Institute for Molecular Medicine and Infectious Disease, Drexel University College of Medicine, Philadelphia, PA, United States; ^3^Department of Neuroscience and Center for Neurovirology, Temple University Lewis Katz School of Medicine, Philadelphia, PA, United States; ^4^Sidney Kimmel Cancer Center, Thomas Jefferson University, Philadelphia, PA, United States

**Keywords:** HIV-1, off-target, CRISPR, unbiased genome-wide, GUIDE-seq, CIRCLE-seq, DISCOVER-seq, whole genome sequencing

## Abstract

As genome-editing nucleases move toward broader clinical applications, the need to define the limits of their specificity and efficiency increases. A variety of approaches for nuclease cleavage detection have been developed, allowing a full-genome survey of the targeting landscape and the detection of a variety of repair outcomes for nuclease-induced double-strand breaks. Each approach has advantages and disadvantages relating to the means of target-site capture, target enrichment mechanism, cellular environment, false discovery, and validation of bona fide off-target cleavage sites in cells. This review examines the strengths, limitations, and origins of the different classes of off-target cleavage detection systems including anchored primer enrichment (GUIDE-seq), *in situ* detection (BLISS), *in vitro* selection libraries (CIRCLE-seq), chromatin immunoprecipitation (ChIP) (DISCOVER-Seq), translocation sequencing (LAM PCR HTGTS), and *in vitro* genomic DNA digestion (Digenome-seq and SITE-Seq). Emphasis is placed on the specific modifications that give rise to the enhanced performance of contemporary techniques over their predecessors and the comparative performance of techniques for different applications. The clinical relevance of these techniques is discussed in the context of assessing the safety of novel CRISPR/Cas9 HIV-1 curative strategies. With the recent success of HIV-1 and SIV-1 viral suppression in humanized mice and non-human primates, respectively, using CRISPR/Cas9, rigorous exploration of potential off-target effects is of critical importance. Such analyses would benefit from the application of the techniques discussed in this review.

## Introduction

Gene-editing strategies involving engineered nucleases [i.e., zinc-finger nucleases (ZFNs), transcription activator-like effector nucleases (TALENs), meganucleases, and clustered regularly interspaced short palindromic repeat (CRISPR) associated nuclease 9 (Cas9)] have made a substantial impact on biological research and offer great therapeutic potential. While CRISPR/Cas9 is the most versatile of these systems it has also exhibited a propensity for off-target activity (Hockemeyer et al., 2011; Mussolino et al., 2011; Cradick et al., 2013; Fu et al., 2013; Hsu et al., 2013; Mali et al., 2013; Pattanayak et al., 2013; Yang et al., 2013; Cho et al., 2014; Lin et al., 2014; Zhang et al., 2014; Liang et al., 2015; Aryal et al., 2018). Understanding and mitigating the off-target activity in the clinical use of gene therapy is of particular importance because off-target effects may not be limited to transient events but may be pertinent to the lifetime of edited cells. Off-target detection methodologies are necessary because the functionality of gene-editing nucleases in general and the CRISPR system in particular are not fully understood. While some studies have indicated that CRISPR is more susceptible to unintended cleavage events than zinc finger nucleases (ZFNs) and transcription activator-like effector nucleases (TALENs), the versatility of CRISPR targeting has rapidly made it the genome editing tool of choice (Deng et al., 2018; Huang et al., 2018; Panfil et al., 2018; Foss et al., 2019; Gao et al., 2019; Karimian et al., 2019; Li et al., 2019). There is data to suggest that the off-target proclivity of CRISPR guide RNAs (gRNAs) can be overcome with proper design considerations (Cho et al., 2014; Dampier et al., 2014, 2017, 2018; Kim et al., 2015; Aryal et al., 2018; Sullivan et al., 2019). Yet the stringent requirements of targeting fidelity that will be necessary to adapt CRISPR systems to their promising range of clinical applications demand a thorough, sensitive survey of the full genomic impact of each gRNA proposed for such applications. Importantly, sensitivity for off-target detection methods is presented as the minimum frequency of occurrence detectable in a cell population. For example, a method that can detect rare off-target events which occur in one out of 1,000 cells is described as having a sensitivity of 0.1%. Sensitivity is discussed in more detail in section Sensitivity.

The variety of published methods for off-target detection each attempt to improve upon earlier methods in some capacity. Trends in improvement include specificity, sensitivity, and throughput, as well as mechanistic considerations such as how off-target cleavage sites are detected and how those sites are enriched for deep sequencing. In this review we have organized our discussion of techniques based on the underlying mechanistic similarities of the assays. It is important to note however, that consideration of other methodological delineations is critical to a complete understanding of the field. In particular a distinction should be made between nomination and validation. Nomination of off-target sites can be achieved *in silico* based on sequence homology or empirically through experimentation. Nomination is important because broad survey of the full genome is necessary to identify where off-target cleavage may occur. Nomination thereby informs validation methods, which are necessarily site-specific, to confirm that off-target cleavage does in fact occur *in cellulo* and *in vivo*. Off-target sites which are validated are commonly referred to as *bona fide* off-target sites.

It is important to note that while this review focuses on underlying mechanism as the basis for grouping techniques for discussion, there is crossover in terms of detected outcomes and downstream utility for some techniques that are presented in separate sections.

Experimental observation of nuclease-induced off-target cleavage falls broadly into two categories termed: biased and unbiased methods. Biased methods make use of *a priori* knowledge to direct site-specific mutation detection and sequence validation to check for mutations at expected off-target sites, i.e., those with high sequence homology to the gRNA (Hsu et al., 2013; Doench et al., 2016; Tsai and Joung, 2016; Aryal et al., 2018). Conversely, unbiased methods are methods that survey the full genome for cleavage events allowing detection of off-target cleavage events independent of predictions (Koo et al., 2015; Tsai and Joung, 2016). While limited, biased techniques are often easier to implement, have a lower cost, or require minimal equipment. In some cases, the ability to rule out predicted, high-potential off-target sites may be enough for experimental purposes. Well-established biased techniques such as T7E1, Surveyor, and targeted amplicon sequencing are also important benchmarks by which newer methods are validated. In some cases, biased techniques generate data that cannot be captured otherwise. Uni-Directional Targeted Sequencing (UDiTaS) for example, requires a known primer site for target enrichment and is capable of detecting translocations, inversions, and large deletions that are missed by other methods (Giannoukos et al., 2018). With the development of the current range of unbiased techniques capable of surveying the full genome, methods relying on *a priori* knowledge play a smaller role. As genome editing becomes increasingly more precise, moving toward a variety of clinical applications, the need to efficiently survey the whole genome for RNA-guided-nuclease target-affinity precludes the use of biased techniques. Although a wide range of unbiased methods have been developed, there is still no clearly-superior, gold-standard technique (note: All off-target detection methods discussed in this manuscript are presented in Table 1 with acronym disambiguation).

**Table 1:** Methods for detection of off-target CRISPR cleavage.

**Method**	**References**	**Acronym disambiguation**	**Description**
Surveyor	Guschin et al., 2010	Surveyor assay	Mismatch cleavage
T7E1	Kim et al., 2009	T7E1 assay	Mismatch cleavage
IDAA	Yang et al., 2015	Indel detection by amplicon analysis	DNA capillary electrophoresis
TIDE	Brinkman et al., 2014	Tracking of indels by decomposition	Indel frequency detection
TIDER	Brinkman et al., 2018	Tracking of insertions, deletions and recombination events	Mutation frequency detection
qEva-CRISPR	Dabrowska et al., 2018	Quantitative evaluation of CRISPR/Cas9-mediated editing	Mutation frequency detection
WGS	Smith et al., 2014; Suzuki et al., 2014; Veres et al., 2014; Iyer et al., 2015	Whole-genome sequencing	Whole genome sequencing
Digenome-seq	Kim et al., 2015	*In vitro* nuclease-digested genome sequencing	*In vitro*, genomic DNA cleavage, WGS
Multiplex Digenome-seq	Kim et al., 2016	Multiplex Digenome-seq	*In vitro*, genomic DNA cleavage, WGS
DIG-Seq	Kim and Kim, 2018	DIG-seq	*In vitro*, genomic DNA cleavage, WGS
SITE-Seq	Cameron et al., 2017	Selective enrichment and identification of tagged genomic DNA ends by sequencing	*In vitro*, genomic DNA cleavage
GOTI	Zuo et al., 2019	Genome-wide off-target analysis by two-cell embryo Injection	*In vivo* cleavage, WGS
*In vitro* selection	Pattanayak et al., 2013	*In vitro* selection with high throughput sequencing	*In vitro*, synthetic library
CIRCLE-seq	Tsai et al., 2017	Circularization for *in vitro* reporting of cleavage effects by sequencing	*In vitro*, genomic library
CHANGE-seq	Lazzarotto et al., 2020	Circularization for high-throughput analysis of nuclease genome-wide effects by sequencing	*In vitro*, genomic library
VIVO	Akcakaya et al., 2018	Verification of *in vivo* off-targets	*In vitro*, genomic library, *in vivo* validation
AMP	Zheng et al., 2014	Anchored multiplex PCR sequencing	Anchored-primer target enrichment
IDLV assay	Wang et al., 2015	Integrase-defective lentiviral vector assay	Anchored-primer target enrichment
GUIDE-seq	Tsai et al., 2015	Genome-wide, unbiased identification of DSBs enabled by sequencing	Anchored-primer target enrichment
iGUIDE	Nobles et al., 2019	Improvement of the GUIDE-seq method	Anchored-primer target enrichment
UDiTaS	Giannoukos et al., 2018	Uni-directional targeted sequencing	Anchored-primer target enrichment
TTISS	Schmid-Burgk et al., 2020	Tagmentation-based tag integration site sequencing	Anchored-primer target enrichment
TC-Seq	Klein et al., 2011	Translocation capture sequencing	Translocation enrichment
HTGTS	Chiarle et al., 2011	High-throughput, genome-wide, translocation sequencing	Translocation enrichment
LAM-PCR HTGTS	Frock et al., 2015; Hu et al., 2016	Linear-amplification-mediated-polymerase chain reaction high-throughput genome-wide translocation sequencing	Translocation enrichment
ChIP-Seq	Iacovoni et al., 2010; Kuscu et al., 2014; Wu et al., 2014; O'Geen et al., 2015	Chromatin immunoprecipitation sequencing	ChIP-seq
DISCOVER-Seq	Wienert et al., 2019, 2020	Discovery of *in situ* Cas off-targets and verification by sequencing	ChIP-seq
BLESS	Crosetto et al., 2013	Direct *in situ* breaks labeling, enrichment on streptavidin and next-generation sequencing	*In situ* end-capture
DSB-Seq	Baranello et al., 2014	Double-strand break sequencing	*In situ* end-capture
END-Seq	Canela et al., 2016	END-Seq	*In situ* end-capture
DSBCapture	Lensing et al., 2016	Double-strand break capture	*In situ* end-capture
BLISS	Yan et al., 2017	Breaks labeling *in situ* and sequencing	*In situ* end-capture
iBLESS	Biernacka et al., 2018	Immobilized-direct *in situ* breaks labeling, enrichment on streptavidin and next-generation sequencing	*In situ* end-capture

## CRISPR/Cas9 Treatment of HIV-1

Gene-editing strategies have the potential to make a significant impact on human immunodeficiency type 1 (HIV-1) treatment. Recent investigations into the application of the CRISPR/Cas9 system have shown potential in using it as a strategy for curing HIV-1 (Dampier et al., 2014, 2017, 2018; Hu et al., 2014; Kaminski et al., 2016a,b,c; Bella et al., 2018; Dash et al., 2019; Kaushik et al., 2019). HIV curative strategies are challenging because of the rapid establishment of a latent reservoir of infected cells (Siliciano and Greene, 2011). During latency, HIV-1 lies dormant and exhibits minimal expression of viral proteins which prevents the immune system from clearing the infection. The reservoir is primarily comprised of CD4+ T cells which can be localized to multiple tissues (Murray et al., 2016). Conventional antiretroviral therapy (ART) cannot remove these latently infected cells, which leads to continuous low levels of viral replication (Blankson et al., 2002). Elimination of HIV DNA from infected individuals remains a challenge in medicine.

There are two approaches to HIV-1 treatment using gene-editing nucleases: targeting the provirus in the latent reservoir and targeting host genes necessary for viral entry into cells. Targeting host genes involves targeting genes for CCR5 and CXCR4, either of which can serve as coreceptors allowing entry of the virus into cells (Hou et al., 2015; Xu et al., 2017; Allen et al., 2018). The goal of this approach is ablation of genes by introduction of insertions or deletions (indels) during endogenous repair processes following nuclease cleavage. Targeting the provirus can have two potentially beneficial outcomes, disruption of viral protein production by introduction of indels into proviral sequence during endogenous repair following nuclease cleavage (Liao et al., 2015; Zhu et al., 2015; Ueda et al., 2016; Wang et al., 2016a,b, 2018; Yoder and Bundschuh, 2016; Mefferd et al., 2018; Ophinni et al., 2018; Roychoudhury et al., 2018) or the excision of the provirus or parts of the provirus *via* simultaneous CRISPR/Cas9 cleavage at two target sites (Ebina et al., 2013; Dampier et al., 2014, 2017; Hu et al., 2014; Kaminski et al., 2016a,b; Yin et al., 2016; Bella et al., 2018). In the context of HIV-1 therapy, the long terminal repeat (LTR) has been identified as a promising target (Liao et al., 2015; Panfil et al., 2018). gRNAs designed to target the HIV-1 5′ (LTR), a region which acts as the HIV-1 promoter, can prevent HIV-1 reactivation by causing either transcriptional silencing or proviral excision because identical LTR sequences bookend the HIV-1 provirus (Kaminski et al., 2016a; Bella et al., 2018; Panfil et al., 2018). Additionally, this type of therapy could have the added benefit of targeting both replication competent and incompetent proviruses, which have the potential of generating viral proteins that are toxic to neighboring cells (Pollack et al., 2017; Baxter et al., 2018). Studies using HIV-1 transgenic mice and humanized mice models revealed that CRISPR-based editing resulted in removal of HIV-1 proviral DNA from several major tissues (Kaminski et al., 2016a; Bella et al., 2018). In another set of experiments, editing of HIV-1 proviral DNA by AAV-CRISPR constructs resulted in complete clearance of replication competent virus from ~40% of animals after the cessation of ART (Dash et al., 2019). In a recent preclinical study, SIV-infected macaques, a well-defined non-human primate model of HIV/AIDS, were treated with AAV9-CRISPR/Cas9 editing constructs targeting LTR and Gag regions of SIV proviral DNA (Mancuso et al., 2020). Remarkably, fragments of integrated SIV proviral DNA were cleaved and removed from viral reservoirs including blood cells and lymphoid tissues leading to a reduction of proviral DNA.

While these observations provide a baseline for the potential use of a CRISPR-based gene editing strategy for the elimination of HIV-1 and a cure of AIDS, evaluation of potential off-target effects becomes highly significant and essential as the field moves closer to clinical translation. The remainder of this review will extensively discuss the landscape of off-target methods that exist today and are commonly used in the field. It will conclude with recommendations for properly assessing the safety of HIV-1 gene therapy.

## Early Techniques for Off-Target Detection Are Biased by a Need for *a priori* Knowledge

The ability to determine off-target cleavage activity of the CRISPR/Cas9 system is crucial for clinical progression of gene editing. While there has been an influx of off-target sequencing assays developed, many publications rely on amplicon sequencing involving PCR amplification of nominated potential off-target sites followed by sequencing to identify off-target cleavage events in selected regions. This method relies on the use of bioinformatic tools to predict potential off-target sites for gRNAs. Using this knowledge, an investigator can extract genomic DNA from treated cells and amplify the regions that were predicted to have an off-target event. The amplified DNA is then checked for any insertion or deletion (indels) events that may have been caused by the CRISPR/Cas9 system. While effective for off-target site validation, genome-wide empirical nomination is still necessary for comprehensive evaluation of targeting specificity.

There are two methods that have risen in popularity to detect off-target events that still rely on PCR but use a different method of detecting indels. These two methods are called the Surveyor and T7E1 assays (Vouillot et al., 2015). In brief, these assays work by hybridizing two pieces of DNA together: an unaltered sample with one that has been mutated by Cas9 or other gene editing proteins. After hybridization of wild type and mutant DNA strands an enzyme is added that recognizes and cleaves bulges or mismatches in the DNA sequence. These enzymes come from bacterial species and are known as resolvases. Once the cleavage reaction has occurred, the digested DNA is run on an agarose gel and banding patterns and band intensities are used to quantitate the levels of gene editing. These assays do not handle single indels well, meaning that identification of a single nucleotide inserted or deleted by Cas9 can be difficult, and they offer no allelic discrimination with respect to editing events.

In order to detect indels, the method of Indel Detection by Amplicon Analysis (IDAA) is a simple yet effective technique which can detect indels with single base pair resolution (Yang et al., 2015; Carballar-Lejarazu et al., 2020). IDAA involves the amplification of potential nuclease cleavage sites using a three-primer amplification which generates fluorescently labeled amplicons. Detection of indels is achieved using DNA capillary electrophoresis. IDAA is considered a simple and effective method for indel detection and quantification of nuclease editing efficiency. Another way to resolve single indels utilizes bioinformatic tools that compare Sanger sequenced samples. One such tool is called tracking of indels by decomposition (TIDE) (Brinkman et al., 2018) and works by aligning unedited sequences with those that have been edited by Cas9. With the two abi trace files and the gRNA, the program finds where that particular gRNA would cleave the DNA and analyzes the peak heights from the chromatograph to determine if there has been an aberrant nucleotide inserted or deleted, indicating editing at that particular location. This tool has limitations when exploring multiple gRNAs and still requires hand-tuning. In order to improve on some of the shortcomings of TIDE, a new tool was developed by Synthego called inference of CRISPR edits (ICE) [https://doi.org/10.1101/251082]. By utilizing techniques from the digital signal-processing field, it de-convolutes overlapping signals in the chromatograph allowing it to detect the composition and frequency of multiple editing events. This adaptation expands the utility to allow multiple gRNAs in a single experiment and rapid batch analysis.

Similar improvements to the TIDE methodology include tracking of insertions, deletions and recombination events (TIDER) and quantitative evaluation of CRISPR/Cas9-mediated editing (qEva-CRISPR) which both allow quantitation of mutation frequency, not limited to indels (Brinkman et al., 2018; Dabrowska et al., 2018). While these tools are mainly used to determine on-target events, they can also be used to measure off-target events. This requires a predictive knowledge of where these off-target events might occur and designing primers to those locations. This represents a serious drawback in the applicability of these tools to detect off-target events. The main reason behind this rationale is the need to design primers targeting suspected sites where Cas9 might bind and cleave. Next-generation sequencing (NGS) data from a number of different techniques has shown that using predicted cut sites will not uncover rare off-target events.

## Gene-Editing Design

### Precise Genome Editing Using RNA-Guided DNA Nuclease Systems

Following the initial discovery of CRISPR/Cas9 system, major adaptations were made to enable the system to work in human cells. These adaptations included: (1) the codon-optimized sequences of Cas9 that ensured preferable expression by the codon table used in the organism of interest (Cong et al., 2013; Jinek et al., 2013) (2) the attached nuclear localization signals (NLSs) to Cas9 to ensure the nuclear localization of Cas9 in human cells (Cong et al., 2013; Jinek et al., 2013), and (3) a single guide RNA (sgRNA), termed gRNA, constructed to possess both the guiding portion in crRNA and an RNA scaffold derived from tracrRNA (Jinek et al., 2012). These adaptations have enabled the CRISPR/Cas9 system to be programmable to any gene region by changing the protospacer sequence and flexible for use in any organism of interest (Hsu et al., 2014).

### Unintentional Cleavage Events Mediated by CRISPR/Cas Nuclease

Evidence of high specificity using nuclease-based genome editing systems is critical for genetic screening in preclinical studies and corresponding transitional research. Since functional DNA is not comprised of random sequence due to evolutionary constraint, identical copies or highly homologous sequences to a designated target could exist in the same genome. Unwanted off-target editing and consequential toxicity has been demonstrated in the use of ZFNs and TALENs (Miller et al., 2007; Szczepek et al., 2007; Guo et al., 2010; Doyon et al., 2011). Soon after the engineered CRISPR/Cas9 was shown to work in human cells, off-target edits induced by CRISPR/Cas9 were addressed using systematic screening approaches (Fu et al., 2013; Hsu et al., 2013; Pattanayak et al., 2013; Qi et al., 2020). Using the Surveyor assay, Cong et al. (2013) showed that some gRNAs bearing up to five mismatches with target sequences induced CRISPR-mediated cleavage. Further experimentation showed that selected gRNAs could induce cleavage events at undesired off-target sites with up to 6 mismatches using the T7E1 assay in three human cell lines. In addition, they did not detect any off-target edits using two selected gRNAs individually at ~50 tested potential sites each. Another study used synthetic oligomers to generate sequence libraries that contained 10^12^ potential off-target sites derived from the sequence of 4 gRNAs (Pattanayak et al., 2013). The results showed that the cleavage events occurred at synthetic off-target sequences with up to 7 mismatches against treated gRNAs, in agreement with previous studies, showing that incomplete complementarity still induced CRISPR-mediated edits. Together these studies suggested several important concepts: (1) the positions of mismatches affected off-target activity; the mismatches distal to PAM site were better tolerated than those proximal to PAM, (2) off-target edits could occur even with more than six mismatches between gRNA and off-target DNA, and (3) design of gRNAs without detectable off-target events is possible; RNF2 and FACNF gRNA caused no off-target mutations.

### Predictive Algorithms for gRNA Selection

The use of computational predictive tools in gRNA design has developed rapidly to accommodate the increasing needs of CRISPR/Cas9 applications. In addition to identifying potential targets, computational tools for gRNA design must rate the exclusivity of those targets in order to avoid the use of gRNAs with off-target proclivity. The search can be as simple as mismatch counts between guide and target. However, recent approaches have adapted sophisticated algorithms into search tools. BLAST serves as the most accessible way to identify off-target sites on the basis of sequence similarity. However, the uniform penalty matrix in BLAST is not sufficient to describe guide-target interaction in the CRISPR/Cas9 system.

Two initial studies utilized similar strategies to characterize off-target activity due to sequence mismatches in the 20-bp complementary target region. One generated a set of gRNA variants that possessed gRNA variants that contained one mismatch against a fixed on-target DNA sequence in the human genome. Hsu et al. quantified the effect of mismatches by high-throughput sequencing of PCR amplicons from the on-target site after treatment of CRISPR/Cas9 (Hsu et al., 2013). Given a 20-bp target site, a set of gRNAs that covered all possible single-mismatch guide sequences were generated systematically such that 3 possible mismatch mutations at each complementary position were synthesized to acquire the contribution of CRISPR/Cas9 activity of each position. The modification frequency at the 20-bp complementary region was used to describe the CRISPR/Cas9 activity at the target site, which was determined by the number of reads that contained either mutations or indels from deep sequencing. The result indicated that the base pairing at the PAM-proximal region tolerated less mismatches than the PAM-distal region. The authors aggregated sequence modification efficiency over 400 gRNA variants from 15 target sites within EMX1 gene regions, which created the pairwise penalty matrix for each type of mismatch spanning the guide-target binding region. A simplified 20-element matrix for a 20 bp guide-target pair was then used as the basis of the scoring algorithm for a gRNA design tool despite the type of mismatches. This score matrix is referred to as the MIT matrix.

Another experimental test using a larger number of gRNA variants demonstrated the improved prediction of potential off-target loci (Doench et al., 2016). The concept remained the same; given a fixed target DNA sequence, the reduction of CRISPR/Cas9 activity due to guide sequence mutations including 1-nucleotide mismatch, 1-nucleotide deletion, and 1-nucleotide insertion was measured. Over 27,000 unique gRNAs were generated to target the coding sequence of human CD33 regardless of PAM alternatives, along with the perfect match gRNAs for each selected target locus. This set of gRNAs gave a high coverage of every mutation type on each of the 20 guide-target base paring as well as every possible PAM. The goal of this experiment was to understand how CRISPR/Cas9 actively disrupts the expression of an easy-to-detect coding gene with or without guide sequence mutations against target DNA. Therefore, reduction of CD33 expression level on the plasma membrane was used to determine the CRISPR/Cas9 activity instead of deep sequencing. The percent activity was calculated by the mean differences of CD33 detected by phycoerythrin-conjugated anti-CD33 antibody between perfect gRNA and variant gRNA. A table of percent activity for every type of mutation (12 mismatch types × 20 positions and 64 possible PAMs) was used to generate the cutting frequency determination (CFD) score. The CFD score of a guide-target pair with multiple mutations is the multiplication of percent activity for specific mutations.

This data along with subsequent data generated from methods discussed below has led to vast increase in the number of computational techniques for predicting the likelihood of off-target cleavage. The range of computational tools for gRNA design includes E-CRISP (Heigwer et al., 2014), CRISPick (Doench et al., 2014), CHOPCHOP (Montague et al., 2014), CRISPR-ERA (Liu et al., 2015), CRISPOR (Haeussler et al., 2016), GUIDES (Meier et al., 2017), GeneArt (Liang et al., 2017), and uCRISPR (Zhang et al., 2019). More recently published tools tend to use the CFD matrix to evaluate penalty scores (i.e., CRISPOR, GUIDES, CRISPick, and GuideScan) and are therefore more reliable tools than those published before the development of the CDF matrix (i.e., E-CRISP, CHOPCHOP, CRISPR-ERA). More recently, the method uCRISPR has been shown to outperform methods using the MIT and CFD matrices (Zhang et al., 2019).

These tools have been reviewed previously and several publications offer a more in-depth review of this topic (Bolukbasi et al., 2016; Tycko et al., 2016; Manghwar et al., 2020; Wang et al., 2020). While the tools are useful for an initial discounting of egregious target choices, *in silico* predictions should always be confirmed by additional techniques.

## Unbiased Techniques

### Whole Genome Sequencing Is a Feasible but Impractical Method for Off-Target Detection

**Figure 1:**
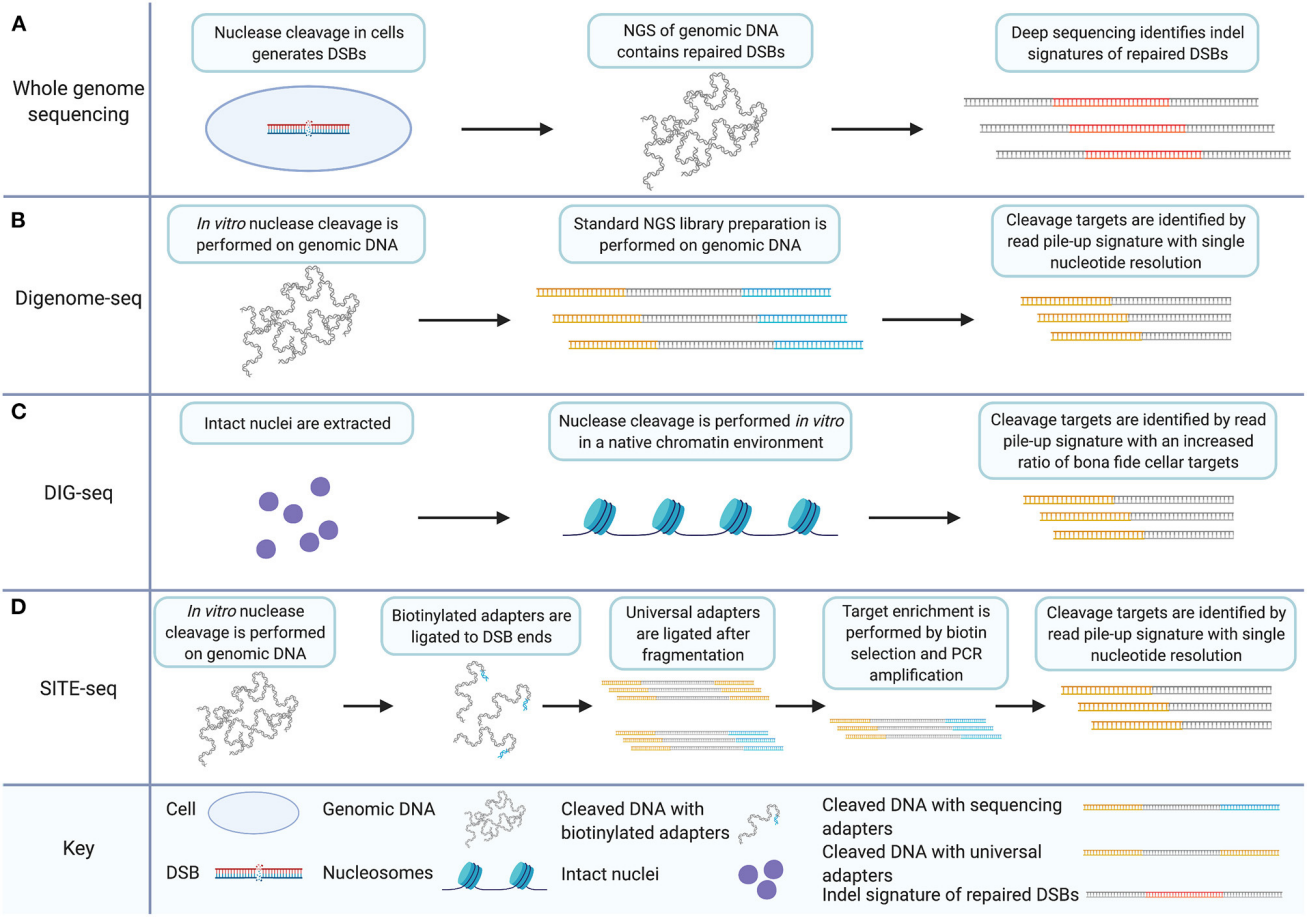
*In vitro* off-target detection methods based on WGS utilize target enrichment strategies that facilitate data analysis and improve signal to noise ratios. **(A)** WGS for CRISPR/Cas9 off-target detection involves nuclease induction of DSBs in cells. Deep sequencing of genomic DNA captures all sequence including indels and genomic rearrangements introduced during DSB repair. **(B)** Digenome-seq involves extraction of genomic DNA and *in vitro* CRISPR/Cas9 treatment which precludes DSB repair. Samples are prepared by standard NGS methods where adapters (in yellow) are added. Cleavage targets are identified by a read pile-up signature with single-nucleotide resolution. **(C)** DIG-seq involves nuclei extraction. Nuclease cleavage is carried out *in vitro* in the native chromatin environment and libraries are prepared for NGS. Cleavage targets are identified by read pile-up, as in standard Digenome-seq. Intact chromatin reduces false discovery and increases the ratio of bona fide cellular targets identified. **(D)** SITE-Seq involves *in vitro* Cas9 digestion, as in Digenome-seq and DIG-seq. Biotinylated adapters (in blue) are then ligated to DSB ends followed by fragmentation and universal adapter ligation. Target enrichment is achieved by biotin selection and PCR amplification. As in Digenome-seq and DIG-seq, cleavage targets are identified by read pile-up during analysis. Created with Biorender.com.

Whole genome sequencing (WGS) is a straightforward approach to unbiased survey of the full genome for off-target nuclease activity. Endogenous repair mechanisms leave sequence-based evidence of nuclease activity on genomic DNA. Non-homologous end-joining (NHEJ) has been shown to introduce indels during the repair of double-strand breaks induced by nucleases (Cradick et al., 2013; Fu et al., 2013; Hsu et al., 2013; Pattanayak et al., 2013). Other repair outcomes for nuclease-induced DSBs include inversions, translocations, and large deletions (Frock et al., 2015; Hu et al., 2016; Giannoukos et al., 2018). Deep sequencing allows the identification of those repaired sites (Figure 1A). WGS ensures a survey of the full genome. There are several advantages to WGS as an off-target detection method. WGS allows an unbiased look at all sites across the genome and has been used to detect unpredicted off-target CRISPR/Cas9 cleavage in clonal cell populations and animal models (Smith et al., 2014; Veres et al., 2014; Dash et al., 2019). WGS detects the behavior of the nucleases in a cellular environment. The signatures of nuclease activity detected by WGS are introduced to the genomic DNA during endogenous repair processes. This is important because cellular features such as chromatin structure have been shown to impact the off-target profile of the CRISPR/Cas9 system (Kuscu et al., 2014; Wu et al., 2014; Chari et al., 2015; Chen et al., 2016, 2017c; Daer et al., 2017; Jensen et al., 2017; Kim and Kim, 2018; Chung et al., 2020). Furthermore, *in vitro* techniques for unbiased off-target detection have demonstrated that CRISPR/Cas9 cleaves more targets *in vitro* compared to targeting within the cellular environment thereby requiring further experimentation to validate the biological relevance of detected targets (Kim et al., 2015; Cameron et al., 2017; Tsai et al., 2017).

WGS has drawbacks though. It is considered inefficient due to a low signal to noise ratio. The vast majority of sequence data collected during WGS represents unedited genomic DNA and the depth-of-coverage for sequence locations of interest is sacrificed to the undisturbed regions. Thus, WGS is limited by throughput, cost, and efficiency compared to whole-genome methods which incorporate target enrichment strategies (e.g., GUIDE-seq) which are discussed in detail later. Nonetheless, the current efficiency of next-generation sequencing does enable this approach. In a study to detect off-target mutations in mice altered with Cas9, a reported 20–25 × depth of coverage was achieved for each sample as a single sequencing library using an Illumina HiSeq 2500 platform (Iyer et al., 2015). Results indicated that a sequencing depth of 10–13X was sufficient to detect 95% of homozygous variants. Other studies report between 33X and 50X coverage as necessary to detect single-nucleotide polymorphisms in human genomes (Bentley et al., 2008; Ajay et al., 2011). Exome sequencing has also been used to assess the targeting specificity of genome editing nucleases (Cho et al., 2014). In a study comparing whole genome sequencing to exome sequencing, the authors conclude that there is no difference in cost effectiveness between the two approaches with respect to detection of known variants across the exome and that WGS produces better uniformity of read coverage. The results of that study show a mean on-target depth of coverage of 14 × to capture 95% of single-nucleotide variants (SNVs) (Meynert et al., 2014).

Modern methods of off-target detection deliver sensitivity on the order of 0.1% meaning that cleavage events which occur in 1 out of 1,000 cells are detectable (Frock et al., 2015; Kim et al., 2015, 2016; Tsai et al., 2015; Cameron et al., 2017; Yan et al., 2017; Kim and Kim, 2018; Wienert et al., 2019). The studies described above do not pinpoint the depth of coverage in WGS necessary to match genome-wide off-target detection methods which incorporate target enrichment strategies. Furthermore, the metrics reported are not directly comparable to off-target detection sensitivity. What those studies indicate is that WGS sensitivity can be variable depending on experimental conditions and sequencing platform and that exome sequencing does not confer an advantage in this strategy.

A recent study which applied WGS for detection of gene-editing outcomes has implemented a technique termed genome-wide off-target analysis by two-cell embryo injection (GOTI) (Zuo et al., 2019). To implement this method single blastomeres of two-cell mouse embryos were edited with CRISPR/cas9 or a base editor and progeny cells were examined by WGS for SNVs. The results of this study showed that CRISRP/Cas9-induced mutations were not carried through cell division, an important characterization of CRISPR/Cas9 effects. GOTI underscores that WGS still plays an important role for off-target detection in some experimental paradigms. Other off-target detection methods would have been unsuitable for collecting these results.

Despite genome-wide surveillance which made WGS a potential choice for off-target detection, recently published methods for unbiased survey of the whole genome offer greater sensitivity, fewer false-positives, and a better signal-to-noise ratio (Frock et al., 2015; Kim et al., 2015; Tsai et al., 2015, 2017; Cameron et al., 2017; Yan et al., 2017; Kim and Kim, 2018; Wienert et al., 2019). For example, the whole genome sequencing approach has been improved in the form of *in vitro* nuclease-digested genome sequencing (Digenome-seq). Digenome-seq enhances WGS performance as an unbiased off-target detection method (Kim et al., 2015, 2016; Kim and Kim, 2018).

### Digenome-seq Enhances WGS Off-Target Detection by Inducing Cleavage *in vitro*

Digenome-seq is an unbiased, *in vitro* off-target cleavage detection technique (Kim et al., 2015). It introduces a change to the WGS approach by implementing nuclease cleavage outside of the cellular environment. Digenome-seq involves the *in vitro* digestion of genomic DNA using CRISPR/Cas9 and the gRNA to be evaluated. The digested genome is then prepared as an ordinary next-generation sequencing library. The alignment of fragment reads from nuclease cleavage sites is distinct from the staggered reads of other fragments because of the absence of sequence repair after nuclease cleavage. This is because endogenous DSBs occur at random locations while the targeted DSBs induced by nuclease cleavage occur at precise sequence locations. Nuclease cleavage sites are distinctly characterized therefore by repeated detection of DSBs at the same sequence location. Digenome-seq achieves target enrichment by introducing a distinct signature to nuclease cleavage targets which improves the resolution of cleavage detection to single-nucleotide precision (Figure 1B). This is not achievable using a WGS approach without the *in vitro* digestion of the genome due to the non-specific nature of the indels relied upon for detection (Kim et al., 2015).

There are several published improvements to the Digenome-seq technique. A multiplex version of Digenome-seq has been published, allowing the testing of multiple gRNAs on the same sample simultaneously (Kim et al., 2016). The multiplex method has several modifications. The algorithm used for analysis was modified to allow the identification of cleavage events that leave different end moieties, specifically one or two nucleotide overhangs; the original algorithm only detected blunt ends. This modification reduced false-negatives and identified targets missed by the original Digenome-seq algorithm. False positives were also reduced compared to Digenome-seq by transcribing gRNAs with a plasmid template rather than an oligonucleotide. Plasmid transcripts were reportedly less heterogenous than oligonucleotide transcripts leading to higher fidelity in target recognition. Multiplex analysis of gRNAs was achievable in the Digenome-seq methodology by choosing gRNAs with target sequences differing by at least 11 nucleotides and thus avoiding ambiguity in target detection within the same sample. Multiplex Digenome-seq results were achieved without an increase in depth of coverage. These results demonstrate not only the ability of the technique to detect off-target cleavage from multiple gRNAs simultaneously but also the ability of Cas9 to be directed to multiple targets *in vitro* by multiplexed gRNAs.

Measures of improvement to off-target detection techniques can depend on the specific measurement goals. The unfettered nature of Digenome-seq with respect to chromatin architecture can be viewed as an advantage compared to WGS or techniques such as genome-wide, unbiased identification of DSBs enabled by sequencing (GUIDE-seq) and high-throughput genome-wide translocation sequencing (HTGTS) (Frock et al., 2015; Tsai et al., 2015). GUIDE-seq and HTGTS are mentioned here to make a point of contrast compared to Digenome-seq; both will be discussed in detail in later sections. The distinction allows for the detection of otherwise obscured gRNA off-target affinities. However, DIG-seq, another Digenome-seq modification, can also be considered an improvement of the Digenome-seq method for the opposite reason (Kim and Kim, 2018). DIG-seq is a Digenome-seq based method applied to DNA with chromatin architecture in place. Native chromatin is isolated *via* nuclei extraction and put through the Digenome-seq protocol (Figure 1C). DIG-seq is considered an improvement over Digenome-seq under the assumption that the cleavage targets that will be detected under these conditions are of keener interest and greater relevance than the full palate of *in vitro* detected cleavage targets outside of the chromatin architecture. This assumption is upheld by the performance of DIG-seq. DIG-seq performance was compared to two other *in vitro* off-target detection methods: selective enrichment and identification of tagged genomic DNA ends by sequencing (SITE-Seq) and circularization for *in vitro* reporting of cleavage effects by sequencing (CIRCLE-seq), discussed in detail below. Although identifying fewer off-target cleavage sites than CIRCLE-seq or SITE-Seq for the same VEGFA target, DIG-seq had a 62% deep sequencing validation rate compared to 29 and 10% validation rate for the other two techniques, respectively.

### SITE-Seq Improves Digenome-seq Methodology With Selective Target Enrichment

SITE-Seq is an unbiased, *in vitro* detection technique for nuclease-induced DSBs (Cameron et al., 2017). SITE-Seq involves the *in vitro* digestion of genomic DNA with CRISPR/Cas9, similar to Digenome-seq. Following 3′ adenylation, DSBs are ligated with biotinylated Illumina-compatible adapters. This leaves a pool of labeled DSBs in genomic DNA, predominately induced by gRNA-guided nuclease cleavage, which allows the selective enrichment of sequence surrounding cleavage sites. Following the initial labeling of the strand break sites, the genomic DNA is fragmented, end-repaired, and 3′ adenylated allowing for another round of Illumina-compatible adapter ligation. Thus, fragments containing sequence from one side of a DSB are exclusively bookended by the P5 and P7 binding-sites necessary for Illumina sequencing. Biotin selection and PCR amplification then lead to a selectively enriched deep-sequencing library comprised predominately of sequences surrounding nuclease cleavage targets.

Similar to Digenome-seq, the technique relies on *in vitro* digestion of genomic DNA and nuclease cleavage targets are distinguished from randomly induced DSBs during sequence analysis by aligned read pileups. SITE-Seq differentiates itself from Digenome-seq particularly by the selective enrichment of nuclease-cleavage targets (Figure 1D). This aspect considerably increases the signal-to-noise ratio of the readout compared to Digenome-seq.

SITE-Seq is highly sensitive, around 0.1%. SITE-Seq analysis of the commonly used controls VEGFA and FANCF detected nearly all of the sites identified by Digenome-seq, GUIDE-seq, and (HTGTS). SITE-Seq reportedly detected all previously identified cellular off-targets from preassembled Cas9-gRNA ribonucleoprotein (RNP). Although Digenome-seq sensitivity is equivalent to SITE-Seq, the signal to noise ratio of SITE-Seq is far greater due to the process of enrichment, which allows sequencing of cleavage sites while excluding the remainder of the genomic DNA. However, SITE-Seq shares the problem of a high false-discovery rate with CIRCLE-seq and Digenome-seq. Cellular factors play a role in the off-target activity of nucleases. *In vitro* techniques identify potential off-target sites in the absence of such factors and the sheer quantity of potential sites can inhibit validation of relevant bona fide sites. For example, SITE-Seq identified nine novel off-target sites for VEGFA and two for FANCF in spite of limiting cellular validation to a subset of identified sites. This is touted as a feature in this instance, and it is a good demonstration of the sensitivity of the method. But if the identified potential off-targets for a particular gRNA are too numerous to be efficiently screened for cellular activity, validation of off-target sites becomes a biased technique in spite of the unbiased nature of the assay. A further complication is that the effect of cellular factors and nuclease concentration on off-target cleavage may limit the relevance of validation to the experimental conditions under which it is carried out.

## *In vitro* Cleavage Libraries

### CIRCLE-seq Is a Highly Sensitive Off-Target Detection Method That Brings Genomic Relevance to *in vitro* Cleavage Libraries

CIRCLE-seq is an unbiased method for detection of off-target CRISPR/Cas9 cleavage (Tsai et al., 2017; Lazzarotto et al., 2018). The method entails fragmentation of genomic DNA *via* sonication, end-repair, and self-ligation of fragments for intramolecular circularization. After circularization, remaining linear DNA is digested using a plasmid-safe ATP-dependent DNase. What remains is a library of circularized fragments of genomic DNA which is then digested using CRISPR/Cas9 and an gRNA to be profiled for off-target affinity. During Cas9 digestion, circles containing on-target and off-target sequence are linearized and are then prepared for next-generation sequencing.

CIRCLE-seq was adapted from earlier published *in vitro* methods for characterizing the off-target profiles of genome-editing nucleases (Pattanayak et al., 2011, 2013). An *in vitro* selection method was introduced to characterize the performance of two zinc-finger nucleases (ZFNs) on a library of 10^11^ sequences. ZFNs targeting human genes for CCR5 and VEGFA were used. VEGFA has become a standard control for evaluation of genome-editing nucleases (Frock et al., 2015; Kim et al., 2015, 2016; Tsai et al., 2015, 2017; Cameron et al., 2017; Yan et al., 2017; Kim and Kim, 2018; Wienert et al., 2019). Both ZFNs were able to cleave target numbers on the order of 10^5^ sequences, the majority of which do not arise in the human genome. CCR5-224 also cleaved 37 *in vitro* human sequence targets 10 of which were validated in human K562 cells. The VEGFA-targeting ZFN, VEGFA2468, cleaved 2652 human sequence targets *in vitro*, 32 of which were validated in human K562 cells (Pattanayak et al., 2011).

**Figure 2:**
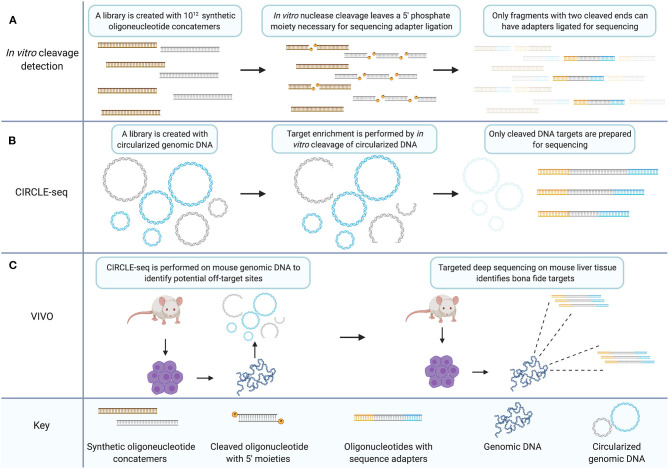
The CIRCLE-seq target enrichment strategy creates an *in vitro* library of CRISPR/Cas9 targets with biological relevance that can inform targeted sequencing for validation of bona fide off-target sites. **(A)** *In vitro* cleavage detection involves generating a library of synthetic oligonucleotide concatemers with potential mismatch targets for a CRISPR gRNA. Target cleavage leaves a 5′ phosphate necessary for ligation of sequencing adapters. Only fragments which have been cleaved at both ends because the target site within the concatemer was recognized by the nuclease two times, are prepared for sequencing. **(B)** CIRCLE-seq involves generation of a circularized *in vitro* library created from genomic DNA. Unrecognized targets remain circularized (in blue) while cleaved target sequences are linearized and can be prepared for sequencing (in gray). **(C)** VIVO involves performing CIRCLE-seq with genomic DNA from mouse primary cells. The CIRCLE-seq results inform targeted deep sequencing with genomic DNA from mouse primary cells derived from CRISPR/Cas9 treated mice thereby validating bona fide off-target sites. Created with Biorender.com.

In a subsequent study, the previous *in vitro* library method for ZFNs was modified to measure CRISPR/Cas9 off-target capacity on an *in vitro* library of 10^12^ sequences (Figure 2A) (Pattanayak et al., 2013). Between two gRNAs tested, five off-target human sequences were validated in HEK293T cells. Both ZFNs and the CRISPR/Cas9 system, were shown to exhibit off-target specificity dependent on enzyme concentration with some rare off-target cleavage events occurring only at higher enzyme concentrations (Pattanayak et al., 2011, 2013).

CIRCLE-seq is a further adaptation of the *in vitro* library off-target cleavage detection method (Figure 2B). Generating the *in vitro* sequence library from genomic DNA increases the relevance of the library of identified cleavage targets. Additionally, because of the mechanism of cleavage-detection in CIRCLE-seq, each readable fragment contains the sequence from both sides of a given cleavage-site allowing for reference-genome-free off-target sequence identification with single nucleotide resolution. Earlier *in vitro* library methods detected significant background sequence noise, with hundreds of thousands of *in vitro* cleavage targets that are not relevant to the human genome. CIRCLE-seq by contrast, finds only human-genome sequence targets.

At the time of initial publication, CIRCLE-seq was the only unbiased, *in vitro* alternative to Digenome-seq and in some facets of performance CIRCLE-seq exceeds Digenome-seq. In particular, CIRCLE-seq has 180,000-fold higher signal-to-noise ratio than Digenome-seq. CIRCLE-seq owes this increase to the process of enrichment which ensures that only cleavage-target sequences are prepared for deep sequencing. There is however a trade-off between the CIRCLE-seq and Digenome-seq techniques in terms of resource consumption as each CIRCLE-seq sample requires 25 μg of genomic DNA while each Digenome-seq sample requires 1 μg. The high background noise in Digenome-seq can make the identification of rare targets difficult, and it has been suggested that some valid off-target cleavage sites are missed by Digenome-seq because of the filtering thresholds necessary to process excessive background signal (Kim et al., 2015; Tsai et al., 2017). CIRCLE-seq is reportedly more sensitive than Digenome-seq. The error rate of current next-generation sequencing (~0.1%) is the limiting factor in the detection of rare off-target cleavage events. Both techniques directly detect cleavage events with single nucleotide resolution which is not common to all off-target detection methods (Frock et al., 2015; Tsai et al., 2015).

Recently an updated version of the CIRCLE-seq methodology has been published (Lazzarotto et al., 2020). The modified technique is called circularization for high-throughput analysis of nuclease genome-wide effects by sequencing (CHANGE-seq). CHANGE-seq utilizes a tagmentation reaction in early steps of the protocol which drastically reduces the labor and preparation time for this methodology. Compared to CIRCLE-seq, CHANGE-seq allows more rapid sample processing for higher-throughput experiments and will likely be the preferred method for any experiment using *in vitro* library digestion in the future.

In comparison to cell-based methods for unbiased off-target detection, *in vitro* methods boast some attractive features. *In vitro* methods avoid the need for transfection, which can complicate both inter- and intra-experimental comparisons. Also *in vitro* detection does not rely on endogenous repair pathways like WGS, GUIDE-seq, and HTGTS (Bentley et al., 2008; Ajay et al., 2011; Meynert et al., 2014; Veres et al., 2014; Frock et al., 2015; Iyer et al., 2015; Tsai et al., 2015). GUIDE-seq and HTGTS are mentioned here to make a point of contrast compared to CIRCLE-seq; both will be discussed in detail in later sections. However, *in vitro* techniques also do not give insight into the behavior of gene-editing nucleases in cells. The false positive rate for CIRCLE-seq is reportedly low enough that the sensitivity limits of deep sequencing inhibit its estimation. However, the false discovery rate is high in CIRCLE-seq meaning that CIRCLE-seq frequently identifies off-target sites *in vitro* that are not validated in cellular experiments.

### VIVO Utilizes CIRCLE-seq to Identify Deep Sequencing Targets for Validation *in vivo*

The standard for validation of bona fide off-target sites is targeted deep sequencing. A method has been published that is termed verification of *in vivo* off-targets (VIVO) which consists of CIRCLE-seq to identify off-target candidate sites followed by targeted deep sequencing to validate those sites (Figure 2C) (Akcakaya et al., 2018). This hybrid technique constitutes a method for validating off-target sites *in vivo* in an animal model. Candidate sites were examined which were identified by CIRCLE-seq in the livers of mice treated with CRISPR/Cas9 in adenoviral vectors using targeted deep sequencing. To do so, they chose a subset of sites from three classes of off-target sequences that they delineate by high, moderate, or low CIRCLE-seq read counts. Results indicate that the probability of validating off-target sites is higher amongst sites that return higher CIRCLE-seq read counts. This agrees with the findings of the originally published CIRCLE-seq method which show that sites with higher CIRCLE-seq read-counts are more likely to be detected by the cell-based method GUIDE-seq (Tsai et al., 2015). Although CIRCLE-seq data sets provide an unbiased genome-wide survey of off-target proclivity for CRISPR/Cas9 gRNAs, the sheer volume of potential off-target sites limited the validation of sites in the VIVO study to a subset of candidates, essentially a biased analysis. Importantly though, off-target sites were validated across all classes in the VIVO study, i.e., high, moderate, and low CIRCLE-seq read counts, underscoring the need for comprehensive analysis of gene-editing nuclease targeting particularly with respect to therapeutic development.

## Anchored Primer Enrichment

### GUIDE-seq Combines the Principles of AMP and IDLV With Improved Off-Target Detection Performance

GUIDE-seq is a method for tagging and enriching the sequence surrounding DSBs for deep sequencing (Tsai et al., 2015). Originally published in 2015, the technique remains an important methodology for assessing the targeting fidelity of genome-editing nucleases (Chaudhari et al., 2020). Briefly, cells are transfected with a plasmid coding for Cas9 and a gRNA and co-transfected with a blunt, double-stranded oligodeoxynucleotide (dsODN). The dsODN is then incorporated into DSBs during NHEJ, thus tagging DSB sites with a short, known sequence. Extracted genomic DNA is then fragmented enzymatically or *via* sonication and the resulting fragments undergo end-repair, dA-tailing, and ligation of a universal adapter sequence which is added to both ends of all fragments. Target enrichment is achieved by two rounds of PCR which amplify only fragments containing the dsODN. Thus, the amplified library consists of strands which each contain one half of the sequence surrounding a DSB repaired by NHEJ. GUIDE-seq is conceptually derived from earlier methods. Precursors to GUIDE-seq include anchored multiplex PCR (AMP) and integrase-defective lentiviral vector (IDLV) integration (Gabriel et al., 2011; Zheng et al., 2014; Wang et al., 2015).

**Figure 3:**
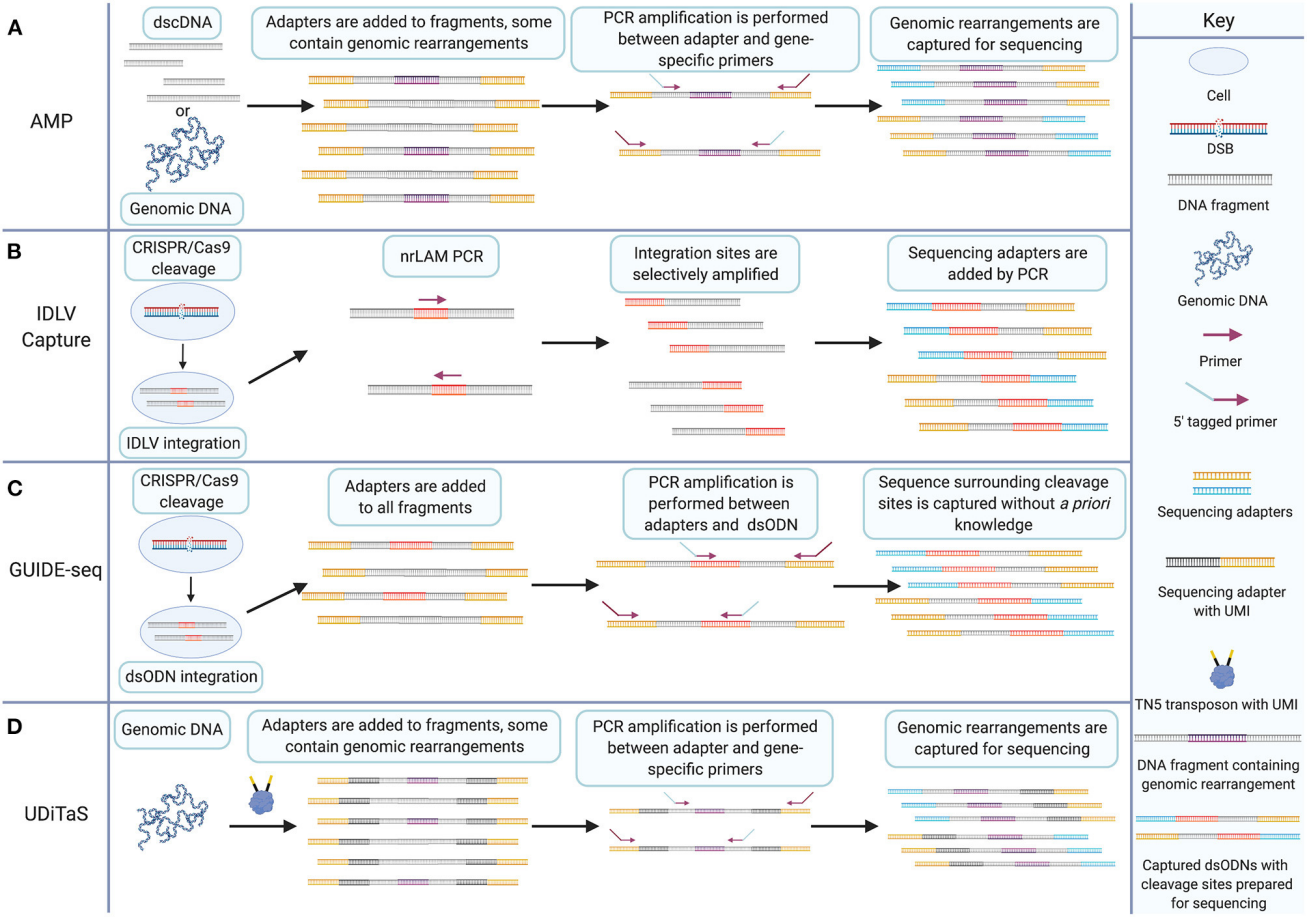
The GUIDE-seq target enrichment strategy combines IDLV capture and AMP for CRISPR/Cas9 cleavage detection without *a priori* knowledge. **(A)** AMP involves adding half-functional adapters (shown in yellow) to both ends of double-stranded cDNA or sheared genomic DNA. Fragments may contain genomic rearrangements that are amplified by PCR between a single anchored gene-specific primer site and a half-functional adapter. 5′ tags on primers enable addition of a second sequencing adapter to amplified target sites. Non-target sites do not get the additional adapter and are excluded from sequencing. **(B)** IDLV capture involves transfection of CRISPR/Cas9 and transduction of IDLV which is integrated into CRISPR/Cas9-induced DSBs during NHEJ, shown in red. nrLAM PCR selectively amplifies cleavage sites from the integrated sequence. Additional rounds of PCR add sequencing adapters (shown in blue and yellow) to the amplicons. **(C)** GUIDE-seq involves transfection of CRISPR/Cas9 and dsODN linkers (shown in red) that are incorporated into cleavage sites during NHEJ. Genomic DNA is fragmented, and half-functional universal adapters (shown in yellow) are added to all fragments. PCR amplification between dsODN and half-functional universal adapters using 5′ tagged primers enables selective amplification of sequence surrounding cleavage sites and the addition of a second adapter necessary for sequencing. **(D)** UDiTaS involves tagmentation of genomic DNA from nuclease-edited cells. Tagmentation fragments DNA and introduces unique molecular indices (UMIs) and adapters. Target enrichment is achieved by selective amplification of fragments between adapters and gene-specific sites. Genomic rearrangements can then be sequence using next-generation sequencing platforms. Created with Biorender.com.

AMP is a target enrichment method for deep sequencing applications. Early target enrichment methods include AmpliSeq, TruSeq Amplicon, HaloPlex, and Nested Patch PCR (Varley and Mitra, 2008; Johansson et al., 2011; Do et al., 2013; Yousem et al., 2013). AMP improves on these techniques by enriching targets with only one known primer binding site rather than two (Figure 3A). In principle, AMP resembles a much earlier method called rapid amplification of cDNA ends (RACE) which utilizes known DNA sequence to determine the sequence of an adjacent region (Frohman et al., 1988). AMP involves preparation of double-stranded cDNA or sheared genomic DNA using earlier published methods (Zheng et al., 2010, 2011; Neiman et al., 2012). Following end-repair and dA-tailing, sequencing adapters, called universal half-functional adapters, are ligated randomly to the ends of all fragments. Enrichment is accomplished by PCR amplification using anchored primers for known targets. Primers for a second round of PCR are 5′-tagged with sequencing adapters. The resulting libraries have a fully functional pair of adapters for deep sequencing. This results in the selective amplification of targets with only one known primer binding site. Unknown adjacent sequence is then captured, and genomic rearrangements can be identified following deep sequencing.

Detection of IDLV integration has been used to identify on- and off-target cleavage of ZFNs, TALENs, and CRISPR/Cas9 (Gabriel et al., 2011; Wang et al., 2015) IDLV detection takes advantage of the IDLV capability to integrate into DSBs during NHEJ. Integration tags break-sites with known sequence which can be exploited for target enrichment (Figure 3B). Targets are amplified for sequencing by linear amplification-mediated (LAM) PCR or non-restrictive LAM (nrLAM) PCR (Schmidt et al., 2007; Gabriel et al., 2009; Paruzynski et al., 2010). IDLV has shortcomings including a low rate of integration and the tendency of IDLVs to sometimes integrate at sites up to 120 bp from the target DSB site (Gabriel et al., 2011; Tsai et al., 2015).

GUIDE-seq technology is a significant advancement over its predecessors. AMP allows the selective amplification of sequence with one side known which was an important step forward from earlier PCR techniques requiring two known primer sites. GUIDE-seq allows selective amplification of a target sequence in which no portion is known by placing the anchor primer on the dsODN (Figure 3C). This is essentially the principle behind IDLV detection but the more reliable rate of uptake of the dsODN into DSBs and the precise integration between the two ends of the DSB mark GUIDE-seq as a significant advance over IDLV.

At the time of publication GUIDE-seq set a new benchmark for off-target detection of nuclease-induced DSBs by filling a methodological gap for unbiased survey of the full genome with an effective target enrichment strategy that greatly improved the signal to noise ratio of off-target detection methods utilizing deep sequencing. GUIDE-seq has a detection sensitivity of ~0.12%, equivalent to that of other current methods (Kim et al., 2015, 2016; Tsai et al., 2015; Cameron et al., 2017; Kim and Kim, 2018). Furthermore, the biological relevance of GUIDE-seq data tends to be more robust than other methods because DSBs are tagged in the context of a cellular environment, not requiring targeted sequence validation for recognition as a *bona fide* editing site.

However, there are several limitations to the GUIDE-seq method. The dsODN, the key component to the effectiveness of the method, has not been adapted to be administered in an animal model, limiting the range of GUIDE-seq application. In addition, the dsODN has shown cytotoxicity in some primary cells (Wienert et al., 2019). Another limitation of GUIDE-seq is its dependence on the endogenous process of NHEJ to detect and tag cleavage events. DSBs not processed by NHEJ will be missed by the GUIDE-seq method.

### iGUIDE Method Reduces Noise in GUIDE-seq Data by Reducing Mispriming Events

A recent update to the GUIDE-seq approach is the iGUIDE method which deals with the problem of mispriming in GUIDE-seq experiments (Nobles et al., 2019). During library preparation, GSP primers can anneal to fragments which lack the dsODN. Amplification can then yield false positive library fragments containing human DNA sequence that were not the sites for nuclease cleavage and dsODN incorporation but functionally resemble true positive library fragments. The iGUIDE method involves the use of a 46 bp dsODN in place of the 34 bp version in the original method. The additional sequence allows filtering of misprimed library fragments during analysis. Use of the iGUIDE method reportedly reveals features of DSB distribution, such as the stronger tendency for spontaneous DSBs to occur near active genes, which are obfuscated by the noise generated by unfiltered mispriming events (Nobles et al., 2019). To date, the iGUIDE method has gained very little traction and is cited by only a single data paper in the literature. Further discussion in this manuscript will be focused on GUIDE-seq in its originally published form.

### TTISS Is a Multiplex GUIDE-seq-Based Method Suitable for Comparison Between Cas9 Variants

Tagmentation-based tag integration site sequencing (TTISS) is a recently published technique which enables a multiplex examination of nucleases and nuclease targets (Schmid-Burgk et al., 2020). The technique is based on GUIDE-seq with some modifications. The protocol is streamlined by utilizing the previously published Tn5 transposase for tagmentation (Picelli et al., 2014). DNA is then purified by spin column and target enrichment is accomplished *via* two nested PCR reactions. TTISS was used to examine the balance between specificity and activity in nine SpCas9 variants including wild-type SpCas9, seven previously published variants, and one novel variant (Kleinstiver et al., 2016; Slaymaker et al., 2016; Chen et al., 2017a; Casini et al., 2018; Hu et al., 2018; Lee et al., 2018; Vakulskas et al., 2018; Schmid-Burgk et al., 2020). The results indicate a trade-off between specificity and activity in general with the precise ratio differing between Cas9 variants. Sequenced targets are attributed to a given Cas9-gRNA pair on the basis of sequence homology. This was effective in the published experiment but could conceivably confound interpretation of some results, limiting the usefulness of TTISS in some contexts. TTISS can reportedly be scaled to accommodate 60 gRNAs per transfection in HEK293T cells. But there is a trade-off in efficiency with 28% fewer off-target sites detected in a multiplexed experiment. The technique is effective for a large-scale screen of Cas9 variants but for a comprehensive look at the full off-target profile of a given Cas9 variant and gRNA-target, the reduced detection efficiency would dictate the use of another technique, e.g., GUIDE-seq or discovery of *in situ* cas off-targets and verification by sequencing (DISCOVER-Seq) (Tsai et al., 2015; Wienert et al., 2019).

### UDiTaS Captures Repair Outcomes Missed by Other Methods but Requires *a priori* Knowledge of Target Sites

GUIDE-seq is not the only relevant modification to the AMP methodology. Uni-directional targeted sequencing (UDiTaS) is also a useful DSB detection technique which utilizes universal adapters and anchored primers to characterize the repair outcomes following engineered nuclease cleavage (Figure 3D) (Giannoukos et al., 2018). The modifications introduced in UDiTaS increase the robustness and utility of the AMP approach. In particular, UDiTaS introduces enzymatic fragmentation known as tagmentation, for genomic DNA rather than shearing by sonication. This modification addresses the tendency for shearing by sonication to introduce damage to genomic DNA that leads to base miscalling during deep sequencing (Costello et al., 2013; Chen et al., 2017b, 2018). UDiTaS introduces a novel Tn5 transposon which contains an Illumina forward adapter (i5), a barcode, and a UMI. Tagmentation yields a fragmented genomic library with adapters on either end of each fragment. Sequence-specific primers are then used to PCR amplify sites targeted by engineered nucleases. A second round of PCR adds an Illumina reverse adapter (i7), similar to the GUIDE-seq protocol. Not only does tagmentation drastically improve efficiency in hands-on time for library preparation protocols, but it also reportedly showed increased library complexity and increased linearity between expected and measured editing outcomes compared to AMP (Giannoukos et al., 2018).

As an off-target detection technique UDiTaS has limited utility due to its biased nature. Sequence-specific primers target sites of interest which require *a priori* knowledge to design. However, UDiTaS has significant utility in its ability to characterize repair outcomes for nuclease-induced cleavage. This is due to the structure of constructed library segments and the use of site-specific primers. Deep sequencing of UDiTaS will capture the junctions of repaired DSBs and thus structural rearrangements can be identified. These include translocations, inversions and large deletions. GUIDE-seq, by its nature does not detect those repair outcomes. The inserted oligonucleotide, which allows anchored priming without sequence knowledge for cleavage sites in GUIDE-seq, allows the capture of only one half of any repaired DSB junction. Reconstruction of complete cleavage sites is accomplished by mapping during analysis (Tsai et al., 2015). Thus, UDiTaS fills an important gap for data relating to repair outcomes for nuclease induced DSBs. Importantly, one approach to the problem of detecting large deletions is to use long read sequencing technologies (Amarasinghe et al., 2020). However, the accuracy and affordability of short-read sequencing platforms by comparison often make short read next-generation sequencing methods preferable and more accessible. An advantage of UDiTas is that it allows the capture and sequencing of large deletions on short read sequencing platforms. Notably, WGS could also be used to detect translocations, inversions, and large deletions but without targeted enrichment the signal to noise ratio of WGS would be markedly lower. Targeted deep sequencing on the other hand cannot capture translocations and efficient capture of inversions and large deletions would require more *a priori* knowledge for targeted deep sequencing than UDiTaS.

## High Throughput Genome-Wide Translocation Sequencing

### HTGTS Is Adapted for Off-Target Detection by Modifications That Enhance Target Enrichment

**Figure 4:**
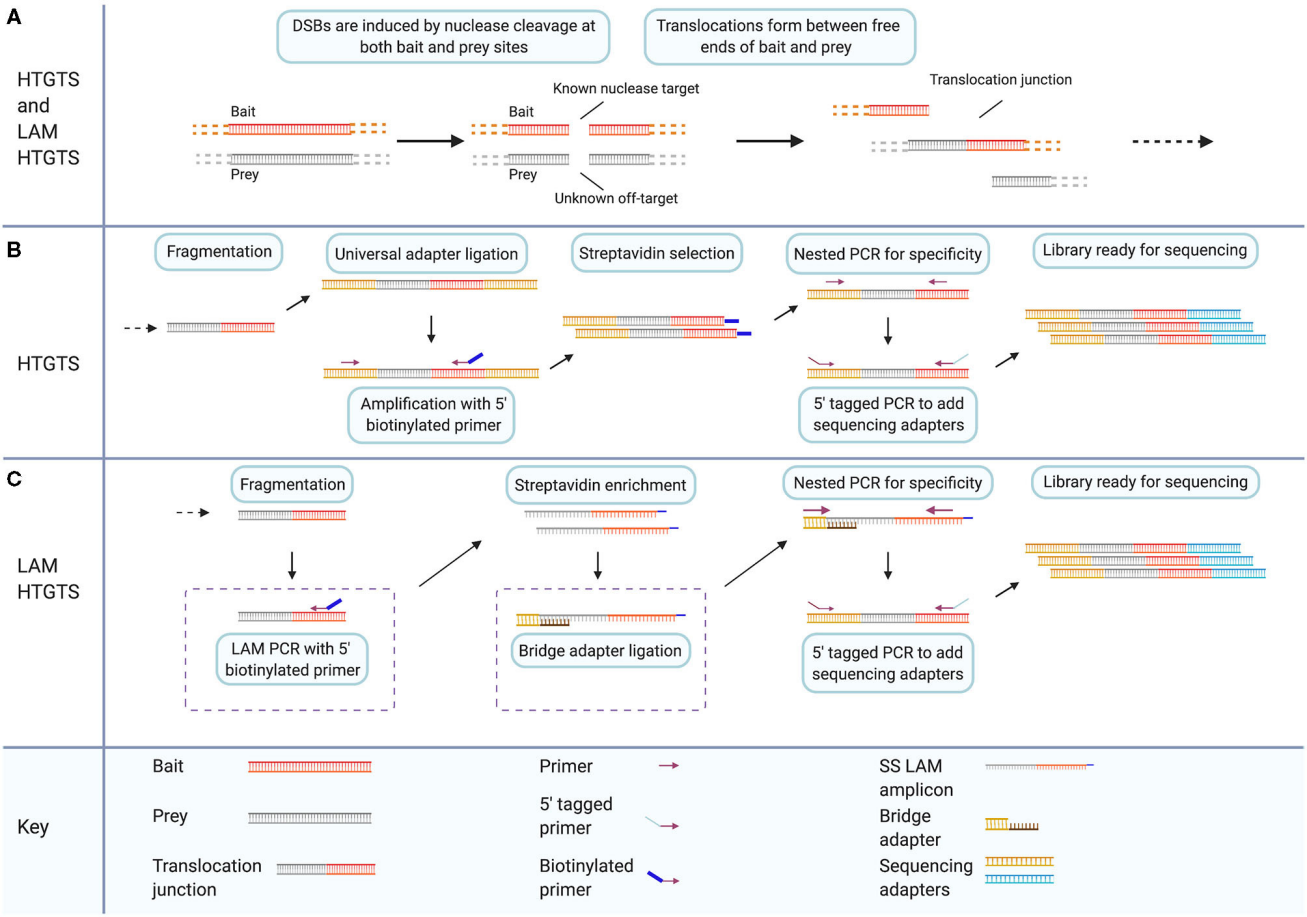
LAM HTGTS has two specific modifications that enhance target enrichment compared to the original method and enable sensitive detection of off-target nuclease cleavage. **(A)** Both HTGTS and LAM HTGTS begin by inducing DSBs through nuclease cleavage in cells for known and unknown sequence targets referred to as bait and prey, respectively, which can form translocation junctions during DSB repair. **(B)** HTGTS involves purification and fragmentation of genomic DNA, ligation of half-functional universal adapters, and PCR amplification of fragments between known bait sequence and universal adapters. Use of a 5′ biotinylated primer during amplification enables Streptavidin enrichment followed by two rounds of PCR for specificity and addition of sequencing adapters. **(C)** LAM HTGTS is similar to the original method with key modifications. One, LAM PCR amplification with 5′ biotinylated primers is followed by Streptavidin enrichment. Two, bridge adapter ligation using and oligo with a 3′ overhang facilitates the further amplification of the single-stranded LAM PCR amplicons which are then prepared for sequencing. Created with Biorender.com.

HTGTS is a method to detect and sequence translocations resulting from DSBs. Originally it was published as a method to study the mechanism of translocation (Chiarle et al., 2011). It has since been adapted as a method to detect off-target cleavage events caused by gene-editing nucleases (Frock et al., 2015). The original published HTGTS method utilized the I-SceI meganuclease to introduce targeted DSBs to specific c-myc and IgH loci. The sites were selected for their frequent involvement in B cell lymphoma oncogenic translocations (Chiarle et al., 2011). DSBs induced at these known locations were then subsequently fused to other DSBs across the genome by endogenous processes (Figure 4A). By exploiting the known sequence of one side of the translocation junction, the sequence of fused sites involved in translocation can then be identified. The original study presented two enrichment chemistries for library preparation to capture the sequence surrounding translocations. Starting with genomic DNA containing translocation fusions with known sequence on one half of the translocation junction, the genomic DNA samples are sheared *via* restriction enzyme digestion. End-repair and adapter ligation are then carried out for all fragments in a sample (Figure 4B).

### LAM HTGTS Adapts HTGTS for Off-Target Detection

The HTGTS method was repurposed for detection of nuclease off-target activity and protocol modifications were introduced that enhance the adapter-PCR target-enrichment methodology of the original method (Figure 4C) (Chiarle et al., 2011; Frock et al., 2015). The modified method is called linear amplification mediated (LAM) high throughput genome-wide translocation sequencing (LAM HTGTS). Applying the HTGTS method, introduced previously, as a nuclease off-target detection method is effectively a function of choosing applicable nucleases to induce desired bait and prey cleavage events. Using the original published method of HTGTS, previously unidentified off-target sites for the I-SceI nuclease were reported. In the updated LAM HTGTS, protocol modifications contribute to the performance of HTGTS as an off-target detection method enabling sensitivity and throughput comparable to other contemporary methods (Frock et al., 2015; Kim et al., 2015, 2016; Tsai et al., 2015; Hu et al., 2016; Cameron et al., 2017; Yan et al., 2017; Kim and Kim, 2018; Wienert et al., 2019).

The two key modifications introduced in the LAM HTGTS protocol are LAM PCR and bridge adapter ligation. LAM PCR is a method of target enrichment for sequences with a single known primer site (Schmidt et al., 2007; Paruzynski et al., 2010). LAM PCR utilizes a 5′ biotinylated primer targeting the known half of each captured junction i.e., one of the two sides of the DSB at the bait site, to linearly amplify across junction sites. Streptavidin selection is then used to magnetically isolate target sequences from genomic DNA. Bridge adapter ligation uses a double-stranded linker with a nucleotide-variable 3′ overhang to facilitate the attachment of adapters to the single-stranded library resulting from linear PCR (Figure 4C) (Zhou et al., 2013; Frock et al., 2015; Hu et al., 2016). Implementing these modifications yields 10–50 times more junctions for sequencing compared to the unmodified HTGTS method (Hu et al., 2016).

Performance of LAM HTGTS is comparable to other methods. For gRNAs targeting VEGFA and EMX1, LAM HTGTS identified the same major off-target sites as GUIDE-seq, although the two methods each identified unique subsets of low frequency off-target cleavage sites. This could be due to the cell lines tested but also to differences in the detection methods, which, by nature, may not be able to identify the same low-abundance cleavage sites (Hu et al., 2016). In particular, HTGTS can capture DSBs containing overhang ends, due to the endogenously repaired nature of translocation junctions, while GUIDE-seq only detects blunt-ended cleavage sites, due to the nature of uptake for oligonucleotide linkers (Tsai et al., 2015; Hu et al., 2016).

One drawback to the LAM HTGTS method is the substantial requirement of starting material. Translocations are rare compared to local rejoining events. They occur in 0.1–0.5% of cells in HTGTS libraries. The authors recommend a starting DNA mass between 20 and 100 μg for a single HTGTS library to achieve a 0.5–1.0 × 10^6^ read depth on an Illumina MiSeq (Hu et al., 2016). GUIDE-seq, by contrast requires 800 ng of genomic DNA to achieve comparable detection sensitivity. Although the authors state that the sensitivity of LAM HTGTS could be increased by starting with even more DNA, the input requirements could be prohibitive for this technique on samples of limited abundance.

There is an additional point worth noting, which is made clear by the results presented in the HTGTS publications (Chiarle et al., 2011; Frock et al., 2015; Hu et al., 2016). Even on-target cleavage events can have undesirable consequences. Translocations contribute to genomic instability (Elliott and Jasin, 2002; Ramiro et al., 2006; Kosicki et al., 2018). Also, translocations can result from on-target cleavage events as readily as off-target cleavage events (Chiarle et al., 2011; Frock et al., 2015; Hu et al., 2016; Kosicki et al., 2018). This point highlights the need for detailed characterization of genome-editing systems.

## Chromatin Immunoprecipitation

### ChIP-seq

DISCOVER-Seq (described below) is an off-target detection method which selectively amplifies CRISPR/Cas9 cleavage sites by detecting the signature of endogenous DNA repair processes (Wienert et al., 2019). The basis of DISCOVER-Seq is ChIP-seq which entails chromatin immunoprecipitation (ChIP) and subsequent deep sequencing of captured DNA fragments (ChIP-seq). Briefly, ChIP begins with formaldehyde crosslinking of a single-cell suspension (Hoffman et al., 2015). Nuclei are then extracted and fragmented *via* sonication. Fragments of interest can then be isolated—pulled down—using bead-bound antibodies allowing the study of protein-DNA interactions (Kim and Ren, 2006; Wienert et al., 2019). In the ChIP-seq methodology, the pulled-down DNA fragments are then prepared for deep sequencing (Wienert et al., 2019).

ChIP has been extensively employed to capture the sequence surrounding DSBs and characterize the genomic landscape of DSBs. Early studies utilized tiled microarrays with DNA pulled down by ChIP in a method dubbed ChIP-chip (Iacovoni et al., 2010; Szilard et al., 2010; Staszewski et al., 2011). More recent studies have moved to ChIP-seq, utilizing contemporary sequencing methods coupled with ChIP (Kim and Ren, 2006; Frietze and Farnham, 2011; Rodriguez et al., 2012; Barlow et al., 2013; Yamane et al., 2013; Zhou et al., 2013; Duan et al., 2014; Kuscu et al., 2014; Wu et al., 2014; Khair et al., 2015; Knight et al., 2015; Madabhushi et al., 2015; O'Geen et al., 2015). γH2AX has been used as a marker for DSBs in ChIP experiments (Iacovoni et al., 2010; Szilard et al., 2010; Rodriguez et al., 2012) DSBs trigger expansive γH2AX binding domains however, and γH2AX can bind kilobases away from the site of a DSB, yielding poor resolution for DSB mapping (Bonner et al., 2008; Iacovoni et al., 2010).

Studies using ChIP-seq to characterize CRISPR/Cas9 off-target proclivity represent early attempts at unbiased survey of Cas9 activity on a genome-wide scale. Multiple studies used ChIP-seq with catalytically inactive Cas9 (dCas9) to pull down Cas9 binding sites (Duan et al., 2014; Kuscu et al., 2014; Wu et al., 2014; Knight et al., 2015; O'Geen et al., 2015). However, ChIP-seq using dCas9 is limited with respect to off-target detection; it has been shown to yield abundant false positives (Kuscu et al., 2014; Wu et al., 2014; Knight et al., 2015; Tsai et al., 2015). For example, only one out of 295 dCas9 binding sites identified by ChIP-seq in mouse embryonic stem cells (mESCs) was identified by targeted sequencing as a bona fide cleavage-target (Wu et al., 2014).

### DISCOVER-Seq Adapts ChIP-seq to an Accurate and Sensitive Off-Target Detection Method Comparable to Other Contemporary Methods

**Figure 5:**
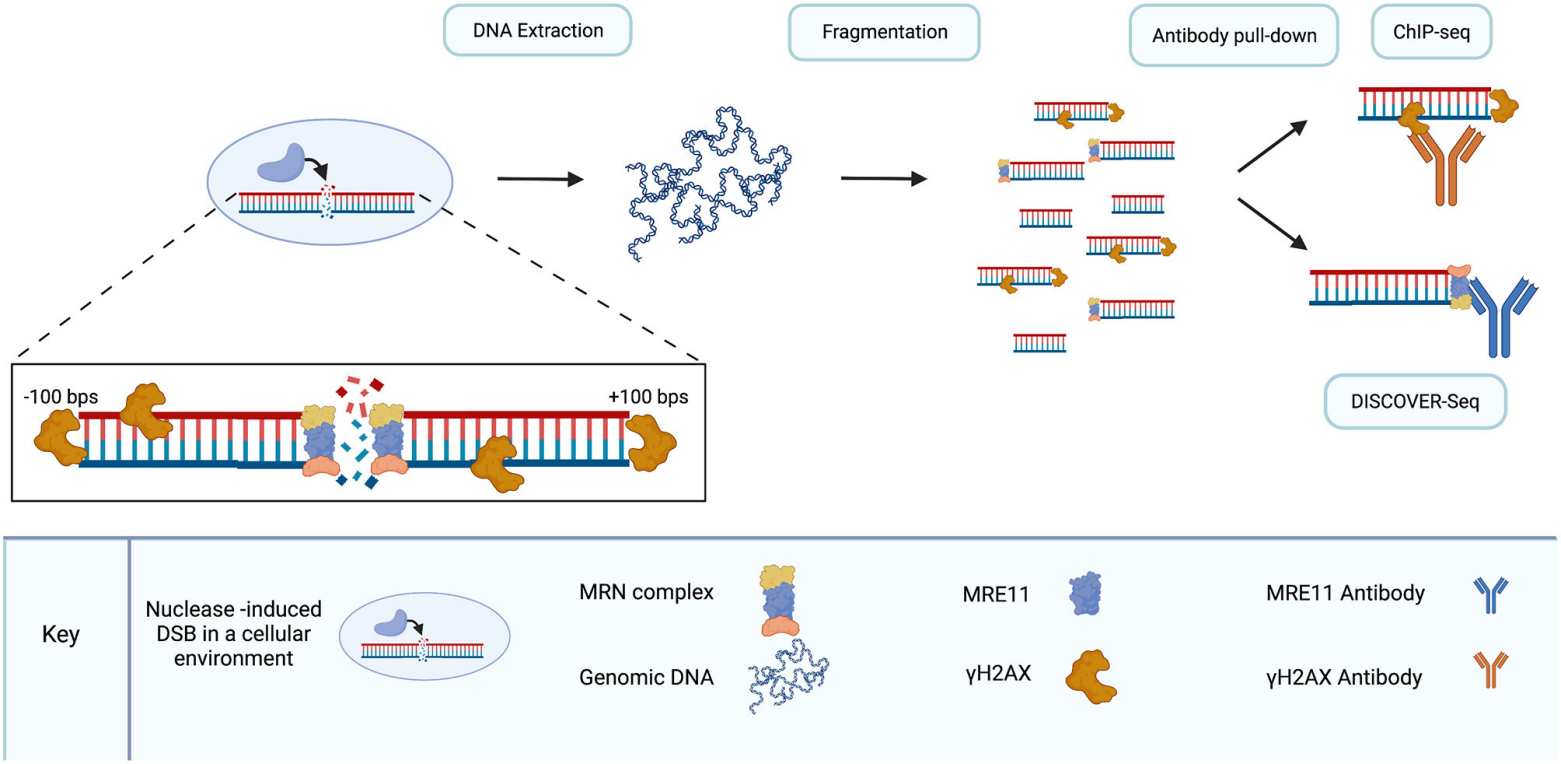
DISCOVER-seq is a specialized version of ChIP-seq. As DSBs are introduced to DNA in living cells, endogenous repair processes recruit proteins to break sites. γH2AX localizes to DSBs within hundreds of base pairs in either direction. The MRN complex, recruited by γH2AX, localizes to the break site. Following genomic DNA extraction and fragmentation, antibody pull-down of fragments enables the sequencing of fragments surrounding DSBs. γH2AX pull-down is used in ChIP-seq and lacks the resolution to precisely locate DSB sites. MRN pull-down, specifically the MRE11 subunit of the MRN complex, enables precise sequencing of DSB sites with single-nucleotide resolution. Use of the MRE11 antibody for pull-down is the distinguishing characteristic of DISCOVER-seq compared to ChIP-seq. Created with Biorender.com.

DISCOVER-Seq advances the ChIP-seq method by utilizing meiotic recombination 11 homolog 1 (MRE11), a DNA repair protein that is part of the MRE11-RAD50-NBS1 (MRN) complex (Figure 5). The MRN complex is involved in DNA damage responses (DDRs) in general, including DSB repair (Connelly and Leach, 2002; Moreno-Herrero et al., 2005; Borde, 2007; Oh and Symington, 2018; Syed and Tainer, 2018; Bian et al., 2019). It also has roles in replication stress, handling of dysfunctional telomeres, cellular response to viral infection, and tumorigenesis (Spehalski et al., 2017; Syed and Tainer, 2018; Bian et al., 2019). Notably, the way that the MRE11 subunit in particular handles different DSB end-moieties may dictate whether DSBs are repaired by HR or NHEJ (Shibata et al., 2014; Liao et al., 2016).

MRE11 is optimal for nuclease-cleavage detection because the MRN complex localizes to DSBs, including those created by CRISPR/Cas9, before ends are joined by repair (Syed and Tainer, 2018; Bian et al., 2019; Wienert et al., 2019). MRN is recruited to DSBs by γH2AX. In addition, MRE-11 is ubiquitous and conserved across all taxonomic kingdoms (Connelly and Leach, 2002; van den Bosch et al., 2003; Wienert et al., 2019). Disruption of each individual component of the MRN complex has been shown to be embryonically lethal in mice (Luo et al., 1999; Zhu et al., 2001; Buis et al., 2008) and mutations in the genes of each individual component have been linked to genomic instability in humans (van den Bosch et al., 2003). Expression of MRE11 across a range of tissues in mice has been demonstrated and following induction of DSBs, MRE11-detection peaks in cells before indels are formed (Wienert et al., 2019).

DISCOVER-Seq detects DSBs with single-nucleotide resolution and compares favorably to other off-target detection methods. However, DISCOVER-Seq reportedly has a sensitivity threshold of 0.3%, slightly higher than other contemporary techniques. A VEGFA target was examined in human K562 cells using both DISCOVER-Seq and GUIDE-seq. They identified 49 off-target sites in common between the techniques but also 41 off-targets sites unique to GUIDE-seq and eight off-target sites unique to DISCOVER-Seq (Wienert et al., 2019). This head-to-head comparison suggests that capture of the entirety of the off-target landscape for at least some gRNAs will require multiple methods. Another favorable feature of DISCOVER-Seq compared to GUIDE-seq is that DISCOVER-Seq works in primary induced pluripotent stem cells (iPSCs). DISCOVER-Seq was shown to detect off-target sites in iPSCs and to differentially detect an allelic specificity in primary cells from a Charcot-Marie-Tooth (CMT) patient with a heterozygous mutation. Data was also shown demonstrating that transfection of the dsODN necessary for GUIDE-seq was toxic to iPSCs (Wienert et al., 2019).

Although other techniques may boast greater sensitivity, DISCOVER-Seq is currently one of only two techniques shown to detect off-target events *in vivo* in an animal model (Wienert et al., 2019); VIVO is the other (Akcakaya et al., 2018). DISCOVER-Seq was tested on the same system as VIVO for comparison. A Pcsk9-gP gRNA was delivered *via* adenoviral infection in a murine model. Mice were then sacrificed at 24-, 26-, and 48-h time points. Twenty-seven off-target sites identified by DISCOVER-Seq were validated by amplicon sequencing and had indel rates between 0.9 and 78.1%. An important point of comparison is that 17 of the 27 sites identified by DISCOVER-Seq were identified by the *in vitro* CIRCLE-seq stage of the VIVO method but were not validated due to the high volume of potential sites generated by the CIRCLE-seq method. This is an important point with respect to the efficiency of *in vitro* techniques and the differential utility of currently available off-target detection methods. Unbiased full-genome survey of the off-target landscape is critical for translation of gene-editing to clinical application. And *in vitro* methods are sensitive and thorough means to characterize the activity of targeted nucleases with respect to sequence homology alone. But the need to validate the high volume of targets detected with *in vitro* methods can lead to a biased survey of high-priority or high-probability sites and bona fide off-target loci can be lost among the false positives.

## *In situ* End-Capture Techniques for Off-Target Detection

*In situ* end-capture methods are a distinct class of techniques which can detect off-target nuclease cleavage by capturing the free ends of DSBs in fixed cells. A variety of *in situ* methods have been published (Crosetto et al., 2013; Baranello et al., 2014; Dorsett et al., 2014; Canela et al., 2016; Lensing et al., 2016; Yan et al., 2017; Biernacka et al., 2018). These methods can be highly sensitive; END-Seq reportedly has a sensitivity of 0.01% and iBLESS can reportedly detect a single DSB in 100,000 cells in *Saccharomyces cerevisiae* (Canela et al., 2016; Biernacka et al., 2018). However, *in situ* methods are limited to the capture of DSBs at a single timepoint preceding cellular response to the induced damage. These methods also tend to have labor intensive protocols with many technical steps. By nature, this class of techniques are less suitable than other methods discussed in this review for research focused on clinical translation of gene editing technologies and more pertinent to studies of enzyme kinetics or the characterization of end moieties following cleavage events. We therefore have reserved an in-depth treatment of this subject for future consideration.

## Comparison Between Methods

To date there is no off-target detection method optimized for all circumstances. Table 2 shows the most relevant modern off-target detection methodologies and the important factors that distinguish each technique. Comparisons between methods rely on gene targets that have been used to evaluate engineered nuclease specificity for years and pre-date the development of unbiased genome-wide techniques. These targets are useful as a metric for comparisons between methods but do not generalize to all anticipated applications of each technology. A recent study compared the performance of GUIDE-seq, CIRCLE-seq, and SITE-Seq side-by-side using promiscuous off-target gRNAs (Chaudhari et al., 2020). Results show that each of the three assays performed with similar efficiency at detection of *bona fide* off-target sites. Results also show that GUIDE-seq has the best correlation of assay signal to observed editing but it is the least reproducible across replicates. Overall, this study concludes that GUIDE-seq is good choice for measuring off-target specificity *ex vivo* in a cellular context but CIRCLE-seq is a good choice for experiments which preclude the use of GUIDE-seq (i.e., studies involving *in vivo* nuclease editing).

**Table 2:** Important features that influence the utility of off-target detection methods.

**Method**	**References**	**Category**	**Sensitivity** ^*^	**Input DNA**	**Detection state**	**Detection mechanism**	**Edits detected**	**Genomic context for nuclease**	**Target enrichment**
Digenome-seq	Kim et al., 2015	*In vitro*	0.1%	1 μg	Target-site DSB	Sequence alignment pile-up	DSBs	Cell free DNA	None
DIG-Seq	Kim and Kim, 2018	*In vitro*	0.1%	1 μg	Target-site DSB	Sequence alignment pile-up	DSBs	Chromatin environment	None
SITE-Seq	Cameron et al., 2017	*In vitro*	0.1%	7.5 μg	Target-site DSB	Sequence alignment pile-up	DSBs	Cell free DNA	Streptavidin selection
CIRCLE-seq	Tsai et al., 2017	*In vitro*	0.01%	25 μg	Target-site DSB	*In vitro* cleavage	DSBs	*In vitro* genomic library	Linearization of circularized library
CHANGE-seq	Lazzarotto et al., 2020	*In vitro*	NR^**^	5 μg	Target-site DSB	*In vitro* cleavage	DSBs	*In vitro* genomic library	Linearization of circularized library
VIVO	Akcakaya et al., 2018	*In vivo*	0.13%	25 μg	Target-site mutation	Targeted sequencing	Repair site mutations (indels)	In organism	Targeted sequencing
GUIDE-seq	Tsai et al., 2015	*Ex vivo*	0.12%	800 ng	Repaired with oligonucleotide incorporation	Oligonucleotide uptake by NHEJ	NHEJ repair sites	Cellular environment	Anchored primer amplification
UDiTaS	Giannoukos et al., 2018	*Ex vivo*	0.1% (all edits), 0.01% (translocations)	50 ng	Repaired with large deletions, inversions, translocations	Targeted sequencing	Repair site mutations (indels), translocations, inversions, large deletions	Cellular environment	Anchored primer amplification
LAM-PCR HTGTS	Frock et al., 2015; Hu et al., 2016	*Ex vivo*	NR^**^	20–100 μg	Translocation junction	Translocation bait and prey	Translocations	Cellular environment	Anchored primer amplification
DISCOVER-seq	Wienert et al., 2019, 2020	*In vivo*	0.3%	2 × 10^6^ – 1 × 10^7^ cells; 40–80 mg homogenized tissue	Unrepaired DSB during DNA damage response (DDR)	MRE11 antibody labeling	Unrepaired DSBs	In organism	ChIP
BLISS	Yan et al., 2017	*In situ*	NR^**^	100 μL of nuclei suspension from cells on 13 mm coverslips	Unrepaired DSB *in situ*	*In situ* end-capture	Unrepaired DSBs	Cellular environment	Transcription

* *Sensitivity is defined as the frequency of occurrence on a per cell basis in a cell population. For example a sensitivity of 0.1% refers to an editing event which occurs in 1 out of 1,000 cells*.

** *Not reported (NR) in the cited manuscript*.

The common thread between all off-target detection methods is that the read-out is always deep sequencing data. Some methods, such as WGS, require more computational post-processing for analysis than others, such as GUIDE-seq or BLISS, which have published analysis pipelines. One point of distinction between methods which may not be readily apparent, is that there is a difference between single-nucleotide resolution in detection and mapping to single-nucleotide resolution during analysis. Digenome-seq and CIRCLE-seq for example, yield sequence data that has single-nucleotide resolution inherent in the DNA library. GUIDE-seq on the other hand, maps to single-nucleotide resolution during data analysis. Another feature of CIRCLE-seq is that it is a reference-genome free method because each fragment in the library contains both ends of the cleavage site.

*In vitro* techniques can be useful in experiments where transfections are difficult and characterization of gene-editing performance independent of endogenous repair pathways is desirable. But the end-goal of experimentation can dictate which method is best on a case-by-case basis. CIRCLE-seq and SITE-Seq are sensitive and thorough, capturing high proportions of potential off-target sites for a given gRNA. They are prone to high false-positive rates, often referred to as false discovery. This is an important distinction. With respect to the *in vitro* off-target detection assay, many of the detected sites are true cuts in the DNA. But they are not bona fide off-target sites which occur in living cells. False discovery is a more apt description for such data points. The high rate of false discovery for these methods may be a drawback in some experimental paradigms where the sheer quantity of data from *in vitro* methods precludes comprehensive validation, thereby requiring a biased follow-up analysis. For example, a subset of off-target sites detected by DISCOVER-Seq were captured by VIVO for the same target but were excluded from the validation set (Akcakaya et al., 2018; Wienert et al., 2019). DIG-Seq is a modification for *in vitro* methods which addresses this problem by maintaining chromatin architecture. The fewer sites identified are therefore more likely to have clinical relevance and accordingly a higher validation rate is reported for DIG-Seq compared to CIRCLE-seq and SITE-Seq (Kim and Kim, 2018).

By contrast, some studies are interested in more than identification of cleavage sites. Repair outcomes are also important. HTGTS and UDiTaS can capture translocations and large genomic rearrangements that are missed by other methods. *In situ* techniques offer a distinctly different strategy that can also be construed as an advantage or disadvantage depending on experimental purpose. Based on a study using H2AX and 53BP1 as DSB markers, the majority of DSBs are resolved within an 8 h timeframe (Asaithamby and Chen, 2009). The *in situ* capture of DSBs at a single timepoint may offer a distinct advantage to enzymology studies whereas the sum total of captured events over time may be of greater interest in other studies.

For off-target detection in animal models, DISCOVER-Seq and VIVO are the best options aside from WGS which has a low signal to noise ratio. While VIVO is more sensitive, DISCOVER-Seq yields a smaller, more clinically relevant data set which may allow an unbiased validation of all identified targets while VIVO may not. However, for off-target detection in a cellular environment, GUIDE-seq is still the most sensitive option which yields the most clinically relevant data. A substantial portion of data (45%) collected by GUIDE-seq was missed by DISCOVER-Seq when looking at the same target. But in some types of primary cells, the dsODN that must be transfected to make GUIDE-seq work, can be cytotoxic (Wienert et al., 2019).

While the different methodologies have distinct mechanisms, there have been several common trends in improvement. Efficiency of each class of technique has been steadily improving. For example, the *in situ* method breaks labeling *in situ* and sequencing (BLISS) is substantially easier and quicker than direct *in situ* breaks labeling, enrichment on streptavidin and next-generation sequencing (BLESS) to carry out without sacrificing sensitivity (Crosetto et al., 2013; Yan et al., 2017). Also, the introduction of Tn5 transposase to replace shearing by sonication has greatly reduced the physical labor involved in library preparation for sequencing. And the sensitivity of all relevant off-target methodologies has been steadily increasing.

## Sensitivity

Sensitivity is an important measure of comparison for assays measuring the same phenomena. An often-described aspect of techniques in terms of sensitivity is detection of a subset of off-target sites that are unique to a particular method when evaluating the same target, i.e., VEGFA or EMX1. But each technique identifies a subset of off-target sites that others do not, and they cannot all be more sensitive than each other. These technique specific subsets are likely due to genomic context or the specific mechanisms of detection and enrichment. Whether or not a technique detects certain off-target sites that other methods miss differs significantly from the explicit definition of sensitivity as the lower limit of frequency in a cell population that can be detected with statistical confidence. For example, as stated earlier, a sensitivity of 0.1% describes an ability to detect events which occur in 1 out of 1,000 cells. The currently competitive and relevant techniques for off-target detection are primarily limited by the error rate of next-generation sequencing techniques not by the inherent capabilities of the assays. Increasing sensitivity in any of these techniques generally requires more starting material and greater sequencing depth. If sequencing depth is the deciding factor in sensitivity, then methods requiring substantially less starting material than others may be distinctly advantageous.

## Throughput of Off-Target Validation Methods

Another area of steady improvement for off-target detection is throughput. This is largely due to improvement in sequencing technology and to target enrichment strategies for off-target cleavage sites. A methodology which is not new but is recently refined and may offer greater throughput for future experiments is rhAmp PCR. rhAmp PCR is used in off-target detection as a validation method that enhances the efficiency and specificity of multiplex PCR by disallowing amplification at sites other than those with exact primer-target homology. Briefly, rhAmp primers require the addition of RNase H2 enzyme to remove a blocking moiety from hybridized primers in order to allow extension. Implementation of rhAmp PCR reduces primer dimers and non-specific amplification (Dobosy et al., 2011). It has been used to facilitate NGS amplicon sequencing allowing higher throughput screening of potential *bona fide* target sites for base editors and CRISPR/Cas9 (Chaudhari et al., 2020; Shapiro et al., 2020; Zeng et al., 2020). Implementation of rhAmp PCR increases the throughput of targeted amplicon screening for bona fide off-target nuclease cleavage.

## Application to the HIV Gene Editing Field

Targeting specificity has been considered in the design of gRNAs targeting HIV (Dampier et al., 2014, 2017, 2018; Hu et al., 2014; Kaminski et al., 2016a,b,c; Wang et al., 2016a,b; Bella et al., 2018; Link et al., 2018; Ophinni et al., 2018; Roychoudhury et al., 2018; Darcis et al., 2019; Sullivan et al., 2019; Chung et al., 2020). Some of the studies investigating HIV-1-CRISPR strategies have examined the off-target activities of gRNAs empirically using biased techniques including T7E1 and Surveyor assays, targeted amplicon sequencing and TIDE (Hou et al., 2015; Ji et al., 2016; Saayman et al., 2016; Yoder and Bundschuh, 2016; Lebbink et al., 2017; Kunze et al., 2018; Ophinni et al., 2018; Wang et al., 2018; Campbell et al., 2019). Other studies have used WGS to analyze the specificity of HIV-targeting gRNAs (Hu et al., 2014; Kaminski et al., 2016a,b,c; Xu et al., 2017; Dash et al., 2019). But rigorous examination of targeting specificity using unbiased, genome-wide techniques has not been applied to HIV-targeting gRNAs to date. For studies that predate 2015, this was unavoidable as most of the unbiased, genome-wide approaches have only been developed recently. However, as gene-editing strategies move closer to developing into viable treatment options, the need for high-throughput off-target screening will play an increasingly important role.

Thus far, the limited application of unbiased, genome-wide off-target detection for HIV-targeting gRNAs has been adequate. Most studies have been focused on the considerable need for establishing a proof of concept for the application of this technology and rigorous off-target analysis has not been of paramount importance in establishing the functional aspects of this approach. For example, some studies have established optimal proviral targets for viral deactivation. The LTR is the most common HIV-1 CRISPR/Cas9 target investigated thus far (Ebina et al., 2013; Dampier et al., 2014, 2017; Hu et al., 2014; Zhu et al., 2015; Bialek et al., 2016; Ji et al., 2016; Kaminski et al., 2016a,b,c; Limsirichai et al., 2016; Saayman et al., 2016; Ueda et al., 2016; Wang et al., 2016a,b; Yin et al., 2016; Lebbink et al., 2017; Zhao et al., 2017; Bella et al., 2018; Kunze et al., 2018; Roychoudhury et al., 2018; Campbell et al., 2019; Darcis et al., 2019; Dash et al., 2019; Kaushik et al., 2019; Su et al., 2020). The ability of CRISPR/Cas9 to deactivate the virus in cell lines, primary cells *ex vivo* and human primary cells in engrafted in mice has also been established (Ebina et al., 2013; Hu et al., 2014; Kaminski et al., 2016a,b; Lebbink et al., 2017; Bella et al., 2018; Ophinni et al., 2018; Campbell et al., 2019; Darcis et al., 2019). Also, the mechanism of that action—mutation, excision, or inversion—has been investigated (Mefferd et al., 2018; Binda et al., 2020). Other studies have characterized viral escape mechanisms and established that a multiplex targeting approach can prevent the emergence of escape mutants (Wang et al., 2016a,b, 2018; Yoder and Bundschuh, 2016; Lebbink et al., 2017; Zhao et al., 2017; Gao et al., 2020). It has also been demonstrated that Tat-driven CRISPR/Cas9 expression can create a negative feedback system that quenches CRISPR/Cas9 production in the absence of viral protein production (Kaminski et al., 2016c). Recently, great strides have been made in demonstrating the utility of the CRISPR/Cas9 system paired with long-acting slow-effective release (LASER) ART in clearing HIV-1 infection from a humanized mouse model (Dash et al., 2019). Additionally, the delivery of CRISPR/Cas9 using AAV vectors has been demonstrated as a viable approach (Kaminski et al., 2016a; Kunze et al., 2018; Dash et al., 2019; Mancuso et al., 2020). Also, an *in vitro* model for magnetically delivering CRISPR/Cas9 across the blood-brain barrier has been developed (Kaushik et al., 2019).

So far, the limited application of off-target analysis has been appropriate to the goals of these proof-of-concept studies. But as CRISPR/Cas9 treatment moves toward clinical application, the gRNAs that are going to be used for clinical treatment will require rigorous off-target analysis. There are published results to uphold this viewpoint. The off-target proclivity of HIV-targeting gRNAs was investigated using targeted amplicon deep-sequencing for the top three off-target candidate sites on each of three gRNAs. No mutations above background level were found at the observed sites. Nonetheless, stable expression of the LTR6 gRNA was found to severely reduce the viability of SupT1 cells (Lebbink et al., 2017). This likely indicates that the off-target screening methodology used was not thorough or sensitive enough to identify all off-target events. These results support the notion that biased targeted examination of potential off-target sites is not sufficient to fully characterize the specificity of gene-editing systems. Results presented in the VIVO and DISCOVER-Seq studies also support this point (Akcakaya et al., 2018; Wienert et al., 2019). DISCOVER-Seq identified bona fide off-target sites for the Pcsk9-gP gRNA that were also identified by VIVO in the *in vitro* CIRCLE-seq phase of the experiment but were not prioritized for further analysis by targeted amplicon sequencing.

As no off-target detection method is ideal in all cases, it is important to consider the factors involved in HIV gene therapy. Table 3 describes a set of criteria for choosing the ideal method at each stage of the development process. As gRNAs are refined and screened for target specificity with the goal of clinical translation, different off-target detection methodologies are best suited for different phases of evaluation. As described earlier, there are two main phases to this process: nomination and validation. Here a further distinction is made and the evaluation process for gRNAs is described in three phases: discovery, refinement, and validation (Table 3). In this paradigm discovery and refinement are two aspects of nomination. In the discovery phase it is important to be able to rapidly and affordably screen potential gRNAs for off-target risks. Computational methods can be employed for this task due to their rapid turn-around time, but moderate false negative rates and high false discovery rates may exclude some good gRNAs. Ideally, SITE-Seq should be used to avoid excluding potentially good candidates. In the refinement stage it is important to have methods that can evaluate the candidates in cells of interest. While DISCOVER-Seq can be used in both *in cellulo* and *in vivo* conditions, it is limited to detecting DSBs that are extant at the time of sampling. With the dynamic nature of these breaks, it is important to understand the accumulated total spectrum of possible targets to produce an appropriate candidate list for validation. GUIDE-seq is the ideal method for this stage. With candidate sites in hand, it is important to validate the entire spectrum of the repair profile in edited cells.

**Table 3:** Criteria for choosing the appropriate off-target detection method for each stage of gRNA development.

	**Acceptable**	**Ideal**
**Discovery** Initial iterations require rapid and low-cost techniques	**Computational Prediction** • Instantaneous • Low cost • High FDR • Moderate FNR	**SITE-Seq** • Rapid • Low cost • High FDR • Low FNR
**Refinement** Candidate gRNA refinement requires a cellular context and should not rely on *a priori* known candidate sites	**DISCOVER-Seq** • Moderate cost • *In vivo* or *in cellulo* cutting context • Only detects extant DSBs	**GUIDE-seq** • Streamlined for throughput • Moderate cost • Low FDR • Low FNR • Detects cumulative DSBs
**Validation** Final safety validation of gRNAs for human trials should be evaluated in animal models as well as *ex vivo* tissue samples using methods that capture the entire repair profile	**Amplicon Sequencing** • Low cost • Low FDR • Low FNR • Misses some repair types	**UDiTaS** • Streamlined for throughput • Moderate cost • Low FDR • Low FNR • Detects all repair types

*Three phases of gRNA evaluation are presented. For each phase an acceptable method and an ideal method are described with a list of primary attributes for each. For acceptable methods, main drawbacks are shown in red. For ideal methods distinguishing advantages are displayed in green*.

In the validation phase it is important to fully characterize the editing profile of the gRNAs at all on- and off-target sites. The best methods to accomplish this are amplicon sequencing and UDiTaS. At this stage, with the range of targeting sites established using a genome-wide unbiased technique (i.e., GUIDE-seq or DISCOVER-Seq), the use of biased methods requiring *a priori* knowledge is warranted. For this purpose, amplicon sequencing is straight-forward and effective. Whereas GUIDE-seq and DISCOVER-Seq can by their nature only capture sites where editing has occurred, amplicon sequencing reveals the outcome of editing events (e.g., indels) or lack thereof. However, UDiTaS presents several advantages over amplicon sequencing. In addition to capturing both edited and unedited sites, UDiTaS incorporates a UMI thereby allowing quantification of editing efficiency sans PCR bias. Furthermore, as HIV-1 excision therapy will likely require multiple gRNAs delivered simultaneously, it is important to screen for large deletions, a difficult feat for standard amplicon sequencing. UDiTaS solves this problem by utilizing one target specific primer and universal adapters allowing it to capture these alternate repair modalities.

## Closing Remarks

The continued development of off-target detection techniques has been a great boon for genome editing. Some studies have found that off-target events are rare in primary cells and animal models, (Smith et al., 2014; Suzuki et al., 2014; Veres et al., 2014; Iyer et al., 2015). And Zuo et al. showed with GOTI that off-targets introduced to a single blastomere in a two-cell mouse embryo are not carried through as cells divide (Zuo et al., 2019). However, these results do not generalize to all gene editing systems or gRNAs. Rather they demonstrate that gene-editing systems have the potential to be highly specific under the proper conditions and provide proof of concept that high-fidelity nuclease targeting can be achieved. But they do not preclude the need for off-target analysis. There is a potent example of gene therapy having serious adverse effects causing lymphocytosis due to an unforeseen translocation event in one patient (Hacein-Bey-Abina et al., 2003). As new gene-editing systems are developed and more gRNAs are designed, they must be tested empirical and they must also be tested in a variety of conditions. CIRCLE-seq identified 55 sites preferentially cleaved depending on cell type due to the presence of SNVs in the protospacer or PAM underscoring this point (Tsai et al., 2017).

At present it is unclear what the full screening regimen should be to rigorously establish a safety profile for a CRISPR/Cas9 therapeutic. The overlapping portions of data sets for off-target techniques that have been examined on common targets such as VEGFA and EMX1 to facilitate comparison are encouraging with respect to the validity of the methods. But each off-target method has also turned up a subset of bona fide off-target sites which were missed by other methods (Frock et al., 2015; Kim et al., 2015; Tsai et al., 2015, 2017; Cameron et al., 2017; Yan et al., 2017; Kim and Kim, 2018; Wienert et al., 2019). A combination of techniques will be necessary to fully characterize the off-target landscape of any gene-editing system. These strategies will also need to be accompanied by cell viability assays to uphold the results of such screening.

## Author Contributions

AA, C-HC, AGA, WD, TG, IS, MN, and BW conceptualized the manuscript, contributed to writing, and made critical revisions. All authors contributed to the article and approved the submitted version.

## Author Disclaimer

The contents of the paper were solely the responsibility of the authors and do not necessarily represent the official views of the NIH.

## Conflict of Interest

The authors declare that the research was conducted in the absence of any commercial or financial relationships that could be construed as a potential conflict of interest.

## Publisher's Note

All claims expressed in this article are solely those of the authors and do not necessarily represent those of their affiliated organizations, or those of the publisher, the editors and the reviewers. Any product that may be evaluated in this article, or claim that may be made by its manufacturer, is not guaranteed or endorsed by the publisher.

## References

[B1] Ajay S. S. Parker S. C. Abaan H. O. Fajardo K. V. Margulies E. H. (2011). Accurate and comprehensive sequencing of personal genomes. Genome Res. 21, 1498–1505. 10.1101/gr.123638.111 21771779 PMC3166834

[B2] Akcakaya P. Bobbin M. L. Guo J. A. Malagon-Lopez J. Clement K. Garcia S. P.. (2018). *In vivo* CRISPR editing with no detectable genome-wide off-target mutations. Nature 561, 416–419. 10.1038/s41586-018-0500-9 30209390 PMC6194229

[B3] Allen A. G. Chung C. H. Atkins A. Dampier W. Khalili K. Nonnemacher M. R.. (2018). Gene editing of HIV-1 co-receptors to prevent and/or cure virus infection. Front. Microbiol. 9:2940. 10.3389/fmicb.2018.02940 30619107 PMC6304358

[B4] Amarasinghe S. L. Su S. Dong X. Zappia L. Ritchie M. E. Gouil Q. (2020). Opportunities and challenges in long-read sequencing data analysis. Genome Biol. 21:30. 10.1186/s13059-020-1935-5 32033565 PMC7006217

[B5] Aryal N. K. Wasylishen A. R. Lozano G. (2018). CRISPR/Cas9 can mediate high-efficiency off-target mutations in mice *in vivo*. Cell Death Dis. 9:1099. 10.1038/s41419-018-1146-0 30368519 PMC6204134

[B6] Asaithamby A. Chen D. J. (2009). Cellular responses to DNA double-strand breaks after low-dose gamma-irradiation. Nucleic Acids Res. 37, 3912–3923. 10.1093/nar/gkp237 19401436 PMC2709554

[B7] Baranello L. Kouzine F. Wojtowicz D. Cui K. Przytycka T. M. Zhao K.. (2014). DNA break mapping reveals topoisomerase II activity genome-wide. Int. J. Mol. Sci. 15, 13111–13122. 10.3390/ijms150713111 25056547 PMC4139894

[B8] Barlow J. H. Faryabi R. B. Callen E. Wong N. Malhowski A. Chen H. T.. (2013). Identification of early replicating fragile sites that contribute to genome instability. Cell 152, 620–632. 10.1016/j.cell.2013.01.006 23352430 PMC3629730

[B9] Baxter A. E. O'Doherty U. Kaufmann D. E. (2018). Beyond the replication-competent HIV reservoir: transcription and translation-competent reservoirs. Retrovirology 15:18. 10.1186/s12977-018-0392-7 29394935 PMC5797386

[B10] Bella R. Kaminski R. Mancuso P. Young W. B. Chen C. Sariyer R.. (2018). Removal of HIV DNA by CRISPR from patient blood engrafts in humanized mice. Mol. Ther. Nucleic Acids 12, 275–282. 10.1016/j.omtn.2018.05.021 30195766 PMC6011019

[B11] Bentley D. R. Balasubramanian S. Swerdlow H. P. Smith G. P. Milton J. Brown C. G.. (2008). Accurate whole human genome sequencing using reversible terminator chemistry. Nature 456, 53–59. 10.1038/nature07517 18987734 PMC2581791

[B12] Bialek J. K. Dunay G. A. Voges M. Schafer C. Spohn M. Stucka R.. (2016). Targeted HIV-1 latency reversal using CRISPR/Cas9-derived transcriptional activator systems. PLoS ONE 11:e0158294. 10.1371/journal.pone.0158294 27341108 PMC4920395

[B13] Bian L. Meng Y. Zhang M. Li D. (2019). MRE11-RAD50-NBS1 complex alterations and DNA damage response: implications for cancer treatment. Mol. Cancer 18:169. 10.1186/s12943-019-1100-5 31767017 PMC6878665

[B14] Biernacka A. Zhu Y. Skrzypczak M. Forey R. Pardo B. Grzelak M.. (2018). i-BLESS is an ultra-sensitive method for detection of DNA double-strand breaks. Commun. Biol 1:181. 10.1038/s42003-018-0165-9 30393778 PMC6208412

[B15] Binda C. S. Klaver B. Berkhout B. Das A. T. (2020). CRISPR-Cas9 dual-gRNA attack causes mutation, excision and inversion of the HIV-1 proviral DNA. Viruses 12:330. 10.3390/v12030330 32197474 PMC7150824

[B16] Blankson J. N. Persaud D. Siliciano R. F. (2002). The challenge of viral reservoirs in HIV-1 infection. Annu. Rev. Med. 53, 557–593. 10.1146/annurev.med.53.082901.104024 11818490

[B17] Bolukbasi M. F. Gupta A. Wolfe S. A. (2016). Creating and evaluating accurate CRISPR-Cas9 scalpels for genomic surgery. Nat. Methods 13, 41–50. 10.1038/nmeth.3684 26716561

[B18] Bonner W. M. Redon C. E. Dickey J. S. Nakamura A. J. Sedelnikova O. A. Solier S.. (2008). GammaH2AX and cancer. Nat. Rev. Cancer 8, 957–967. 10.1038/nrc2523 19005492 PMC3094856

[B19] Borde V. (2007). The multiple roles of the Mre11 complex for meiotic recombination. Chromosome Res. 15, 551–563. 10.1007/s10577-007-1147-9 17674145

[B20] Brinkman E. K. Chen T. Amendola M. van Steensel B. (2014). Easy quantitative assessment of genome editing by sequence trace decomposition. Nucleic Acids Res. 42:e168. 10.1093/nar/gku936 25300484 PMC4267669

[B21] Brinkman E. K. Kousholt A. N. Harmsen T. Leemans C. Chen T. Jonkers J.. (2018). Easy quantification of template-directed CRISPR/Cas9 editing. Nucleic Acids Res. 46:e58. 10.1093/nar/gky164 29538768 PMC6007333

[B22] Buis J. Wu Y. Deng Y. Leddon J. Westfield G. Eckersdorff M.. (2008). Mre11 nuclease activity has essential roles in DNA repair and genomic stability distinct from ATM activation. Cell 135, 85–96. 10.1016/j.cell.2008.08.015 18854157 PMC2645868

[B23] Cameron P. Fuller C. K. Donohoue P. D. Jones B. N. Thompson M. S. Carter M. M.. (2017). Mapping the genomic landscape of CRISPR-Cas9 cleavage. Nat. Methods 14, 600–606. 10.1038/nmeth.4284 28459459

[B24] Campbell L. A. Coke L. M. Richie C. T. Fortuno L. V. Park A. Y. Harvey B. K. (2019). Gesicle-mediated delivery of CRISPR/Cas9 ribonucleoprotein complex for inactivating the HIV provirus. Mol. Ther. 27, 151–163. 10.1016/j.ymthe.2018.10.002 30389355 PMC6318701

[B25] Canela A. Sridharan S. Sciascia N. Tubbs A. Meltzer P. Sleckman B. P.. (2016). DNA breaks and end resection measured genome-wide by end sequencing. Mol. Cell 63, 898–911. 10.1016/j.molcel.2016.06.034 27477910 PMC6299834

[B26] Carballar-Lejarazu R. Kelsey A. Pham T. B. Bennett E. P. James A. A. (2020). Digital droplet PCR and IDAA for the detection of CRISPR indel edits in the malaria species Anopheles stephensi. BioTechniques 68, 172–179. 10.2144/btn-2019-0103 32040336 PMC7177198

[B27] Casini A. Olivieri M. Petris G. Montagna C. Reginato G. Maule G.. (2018). A highly specific SpCas9 variant is identified by *in vivo* screening in yeast. Nat. Biotechnol. 36, 265–271. 10.1038/nbt.4066 29431739 PMC6066108

[B28] Chari R. Mali P. Moosburner M. Church G. M. (2015). Unraveling CRISPR-Cas9 genome engineering parameters via a library-on-library approach. Nat. Methods 12, 823–826. 10.1038/nmeth.3473 26167643 PMC5292764

[B29] Chaudhari H. G. Penterman J. Whitton H. J. Spencer S. J. Flanagan N. Lei Zhang M. C.. (2020). Evaluation of homology-independent CRISPR-Cas9 off-target assessment methods. CRISPR J 3, 440–453. 10.1089/crispr.2020.0053 33346710 PMC7757695

[B30] Chen J. S. Dagdas Y. S. Kleinstiver B. P. Welch M. M. Sousa A. A. Harrington L. B.. (2017a). Enhanced proofreading governs CRISPR-Cas9 targeting accuracy. Nature 550, 407–410. 10.1038/nature24268 28931002 PMC5918688

[B31] Chen L. Liu P. Evans T. C. Jr. Ettwiller L. M. (2017b). DNA damage is a pervasive cause of sequencing errors, directly confounding variant identification. Science 355, 752–756. 10.1126/science.aai8690 28209900

[B32] Chen L. Liu P. Evans T. C. Jr. Ettwiller L. M. (2018). Response to comment on “DNA damage is a pervasive cause of sequencing errors, directly confounding variant identification”. Science 361. 10.1126/science.aat0958 30262470

[B33] Chen X. Liu J. Janssen J. M. Goncalves M. (2017c). The chromatin structure differentially impacts high-specificity CRISPR-Cas9 nuclease strategies. Mol. Ther. Nucleic Acids 8, 558–563. 10.1016/j.omtn.2017.08.005 28918055 PMC5577405

[B34] Chen X. Rinsma M. Janssen J. M. Liu J. Maggio I. Goncalves M. A. (2016). Probing the impact of chromatin conformation on genome editing tools. Nucleic Acids Res. 44, 6482–6492. 10.1093/nar/gkw524 27280977 PMC5291272

[B35] Chiarle R. Zhang Y. Frock R. L. Lewis S. M. Molinie B. Ho Y. J.. (2011). Genome-wide translocation sequencing reveals mechanisms of chromosome breaks and rearrangements in B cells. Cell 147, 107–119. 10.1016/j.cell.2011.07.049 21962511 PMC3186939

[B36] Cho S. W. Kim S. Kim Y. Kweon J. Kim H. S. Bae S.. (2014). Analysis of off-target effects of CRISPR/Cas-derived RNA-guided endonucleases and nickases. Genome Res. 24, 132–141. 10.1101/gr.162339.113 24253446 PMC3875854

[B37] Chung C. H. Allen A. G. Sullivan N. T. Atkins A. Nonnemacher M. R. Wigdahl B.. (2020). Computational analysis concerning the impact of DNA accessibility on CRISPR-Cas9 cleavage efficiency. Mol. Ther. 28, 19–28. 10.1016/j.ymthe.2019.10.008 31672284 PMC6953893

[B38] Cong L. Ran F. A. Cox D. Lin S. Barretto R. Habib N.. (2013). Multiplex genome engineering using CRISPR/Cas systems. Science 339, 819–823. 10.1126/science.1231143 23287718 PMC3795411

[B39] Connelly J. C. Leach D. R. (2002). Tethering on the brink: the evolutionarily conserved Mre11-Rad50 complex. Trends Biochem. Sci. 27, 410–418. 10.1016/S0968-0004(02)02144-8 12151226

[B40] Costello M. Pugh T. J. Fennell T. J. Stewart C. Lichtenstein L. Meldrim J. C.. (2013). Discovery and characterization of artifactual mutations in deep coverage targeted capture sequencing data due to oxidative DNA damage during sample preparation. Nucleic Acids Res. 41:e67. 10.1093/nar/gks1443 23303777 PMC3616734

[B41] Cradick T. J. Fine E. J. Antico C. J. Bao G. (2013). CRISPR/Cas9 systems targeting beta-globin and CCR5 genes have substantial off-target activity. Nucleic Acids Res. 41, 9584–9592. 10.1093/nar/gkt714 23939622 PMC3814385

[B42] Crosetto N. Mitra A. Silva M. J. Bienko M. Dojer N. Wang Q.. (2013). Nucleotide-resolution DNA double-strand break mapping by next-generation sequencing. Nat. Methods 10, 361–365. 10.1038/nmeth.2408 23503052 PMC3651036

[B43] Dabrowska M. Czubak K. Juzwa W. Krzyzosiak W. J. Olejniczak M. Kozlowski P. (2018). qEva-CRISPR: a method for quantitative evaluation of CRISPR/Cas-mediated genome editing in target and off-target sites. Nucleic Acids Res. 46:e101. 10.1093/nar/gky505 29878242 PMC6158505

[B44] Daer R. M. Cutts J. P. Brafman D. A. Haynes K. A. (2017). The impact of chromatin dynamics on Cas9-mediated genome editing in human cells. ACS Synth. Biol. 6, 428–438. 10.1021/acssynbio.5b00299 27783893 PMC5357160

[B45] Dampier W. Nonnemacher M. R. Sullivan N. T. Jacobson J. M. Wigdahl B. (2014). HIV excision utilizing CRISPR/Cas9 technology: attacking the proviral quasispecies in reservoirs to achieve a cure. MOJ Immunol. 1. 10.15406/moji.2014.01.00022 25893217 PMC4399856

[B46] Dampier W. Sullivan N. T. Chung C. H. Mell J. C. Nonnemacher M. R. Wigdahl B. (2017). Designing broad-spectrum anti-HIV-1 gRNAs to target patient-derived variants. Sci. Rep. 7:14413. 10.1038/s41598-017-12612-z 29089503 PMC5663707

[B47] Dampier W. Sullivan N. T. Mell J. C. Pirrone V. Ehrlich G. C.. (2018). Broad spectrum and personalized gRNAs for CRISPR/Cas9 HIV-1 therapeutics. AIDS Res. Hum. Retroviruses. 34, 950–960. 10.1089/aid.2017.0274 29968495 PMC6238604

[B48] Darcis G. Binda C. S. Klaver B. Herrera-Carrillo E. Berkhout B. Das A. T. (2019). The impact of HIV-1 genetic diversity on CRISPR-Cas9 antiviral activity and viral escape. Viruses 11. 10.3390/v11030255 30871200 PMC6466431

[B49] Dash P. K. Kaminski R. Bella R. Su H. Mathews S. Ahooyi T. M.. (2019). Sequential LASER ART and CRISPR treatments eliminate HIV-1 in a subset of infected humanized mice. Nat. Commun. 10:2753. 10.1038/s41467-019-10366-y 31266936 PMC6606613

[B50] Deng Q. Chen Z. Shi L. Lin H. (2018). Developmental progress of CRISPR/Cas9 and its therapeutic applications for HIV-1 infection. Rev. Med. Virol. 28:e1998. 10.1002/rmv.1998 30024073

[B51] Do H. Wong S. Q. Li J. Dobrovic A. (2013). Reducing sequence artifacts in amplicon-based massively parallel sequencing of formalin-fixed paraffin-embedded DNA by enzymatic depletion of uracil-containing templates. Clin. Chem. 59, 1376–1383. 10.1373/clinchem.2012.202390 23649127

[B52] Dobosy J. R. Rose S. D. Beltz K. R. Rupp S. M. Powers K. M. Behlke M. A.. (2011). RNase H-dependent PCR (rhPCR): improved specificity and single nucleotide polymorphism detection using blocked cleavable primers. BMC Biotechnol. 11:80. 10.1186/1472-6750-11-80 21831278 PMC3224242

[B53] Doench J. G. Fusi N. Sullender M. Hegde M. Vaimberg E. W. Donovan K. F.. (2016). Optimized sgRNA design to maximize activity and minimize off-target effects of CRISPR-Cas9. Nat. Biotechnol. 34, 184–191. 10.1038/nbt.3437 26780180 PMC4744125

[B54] Doench J. G. Hartenian E. Graham D. B. Tothova Z. Hegde M. Smith I.. (2014). Rational design of highly active sgRNAs for CRISPR-Cas9-mediated gene inactivation. Nat. Biotechnol. 32, 1262–1267. 10.1038/nbt.3026 25184501 PMC4262738

[B55] Dorsett Y. Zhou Y. Tubbs A. T. Chen B. R. Purman C. Lee B. S.. (2014). HCoDES reveals chromosomal DNA end structures with single-nucleotide resolution. Mol. Cell 56, 808–818. 10.1016/j.molcel.2014.10.024 25435138 PMC4272619

[B56] Doyon Y. Vo T. D. Mendel M. C. Greenberg S. G. Wang J. Xia D. F.. (2011). Enhancing zinc-finger-nuclease activity with improved obligate heterodimeric architectures. Nat. Methods 8, 74–79. 10.1038/nmeth.1539 21131970

[B57] Duan J. Lu G. Xie Z. Lou M. Luo J. Guo L.. (2014). Genome-wide identification of CRISPR/Cas9 off-targets in human genome. Cell Res. 24, 1009–1012. 10.1038/cr.2014.87 24980957 PMC4123298

[B58] Ebina H. Misawa N. Kanemura Y. Koyanagi Y. (2013). Harnessing the CRISPR/Cas9 system to disrupt latent HIV-1 provirus. Sci. Rep. 3:2510. 10.1038/srep02510 23974631 PMC3752613

[B59] Elliott B. Jasin M. (2002). Double-strand breaks and translocations in cancer. Cell. Mol. Life Sci. 59, 373–385. 10.1007/s00018-002-8429-3 11915950 PMC11146114

[B60] Foss D. V. Hochstrasser M. L. Wilson R. C. (2019). Clinical applications of CRISPR-based genome editing and diagnostics. Transfusion 59, 1389–1399. 10.1111/trf.15126 30600536

[B61] Frietze S. Farnham P. J. (2011). Transcription factor effector domains. Subcell. Biochem. 52, 261–277. 10.1007/978-90-481-9069-0_12 21557087 PMC4151296

[B62] Frock R. L. Hu J. Meyers R. M. Ho Y. J. Kii E. Alt F. W. (2015). Genome-wide detection of DNA double-stranded breaks induced by engineered nucleases. Nat. Biotechnol. 33, 179–186. 10.1038/nbt.3101 25503383 PMC4320661

[B63] Frohman M. A. Dush M. K. Martin G. R. (1988). Rapid production of full-length cDNAs from rare transcripts: amplification using a single gene-specific oligonucleotide primer. Proc. Natl. Acad. Sci. U.S.A. 85, 8998–9002. 10.1073/pnas.85.23.8998 2461560 PMC282649

[B64] Fu Y. Foden J. A. Khayter C. Maeder M. L. Reyon D. Joung J. K.. (2013). High-frequency off-target mutagenesis induced by CRISPR-Cas nucleases in human cells. Nat. Biotechnol. 31, 822–826. 10.1038/nbt.2623 23792628 PMC3773023

[B65] Gabriel R. Eckenberg R. Paruzynski A. Bartholomae C. C. Nowrouzi A. Arens A.. (2009). Comprehensive genomic access to vector integration in clinical gene therapy. Nat. Med. 15, 1431–1436. 10.1038/nm.2057 19966782

[B66] Gabriel R. Lombardo A. Arens A. Miller J. C. Genovese P. Kaeppel C.. (2011). An unbiased genome-wide analysis of zinc-finger nuclease specificity. Nat. Biotechnol. 29, 816–823. 10.1038/nbt.1948 21822255

[B67] Gao Q. Dong X. Xu Q. Zhu L. Wang F. Hou Y.. (2019). Therapeutic potential of CRISPR/Cas9 gene editing in engineered T-cell therapy. Cancer Med. 8, 4254–4264. 10.1002/cam4.2257 31199589 PMC6675705

[B68] Gao Z. Fan M. Das A. T. Herrera-Carrillo E. Berkhout B. (2020). Extinction of all infectious HIV in cell culture by the CRISPR-Cas12a system with only a single crRNA. Nucleic Acids Res. 48, 5527–5539. 10.1093/nar/gkaa226 32282899 PMC7261156

[B69] Giannoukos G. Ciulla D. M. Marco E. Abdulkerim H. S. Barrera L. A. Bothmer A.. (2018). UDiTaS, a genome editing detection method for indels and genome rearrangements. BMC Genomics 19:212. 10.1186/s12864-018-4561-9 29562890 PMC5861650

[B70] Guo J. Gaj T. Barbas C. F. 3rd (2010). Directed evolution of an enhanced and highly efficient FokI cleavage domain for zinc finger nucleases. J. Mol. Biol. 400, 96–107. 10.1016/j.jmb.2010.04.060 20447404 PMC2885538

[B71] Guschin D. Y. Waite A. J. Katibah G. E. Miller J. C. Holmes M. C. Rebar E. J. (2010). A rapid and general assay for monitoring endogenous gene modification. Methods Mol. Biol. 649, 247–256. 10.1007/978-1-60761-753-2_15 20680839

[B72] Hacein-Bey-Abina S. von Kalle C. Schmidt M. Le Deist F. Wulffraat N. McIntyre E.. (2003). A serious adverse event after successful gene therapy for X-linked severe combined immunodeficiency. N. Engl. J. Med. 348, 255–256. 10.1056/NEJM200301163480314 12529469

[B73] Haeussler M. Schonig K. Eckert H. Eschstruth A. Mianne J. Renaud J. B.. (2016). Evaluation of off-target and on-target scoring algorithms and integration into the guide RNA selection tool CRISPOR. Genome Biol. 17:148. 10.1186/s13059-016-1012-2 27380939 PMC4934014

[B74] Heigwer F. Kerr G. Boutros M. (2014). E-CRISP: fast CRISPR target site identification. Nat. Methods 11, 122–123. 10.1038/nmeth.2812 24481216

[B75] Hockemeyer D. Wang H. Kiani S. Lai C. S. Gao Q. Cassady J. P.. (2011). Genetic engineering of human pluripotent cells using TALE nucleases. Nat. Biotechnol. 29, 731–734. 10.1038/nbt.1927 21738127 PMC3152587

[B76] Hoffman E. A. Frey B. L. Smith L. M. Auble D. T. (2015). Formaldehyde crosslinking: a tool for the study of chromatin complexes. J. Biol. Chem. 290, 26404–26411. 10.1074/jbc.R115.651679 26354429 PMC4646298

[B77] Hou P. Chen S. Wang S. Yu X. Chen Y. Jiang M.. (2015). Genome editing of CXCR4 by CRISPR/cas9 confers cells resistant to HIV-1 infection. Sci. Rep. 5:15577. 10.1038/srep15577 26481100 PMC4612538

[B78] Hsu P. D. Lander E. S. Zhang F. (2014). Development and applications of CRISPR-Cas9 for genome engineering. Cell 157, 1262–1278. 10.1016/j.cell.2014.05.010 24906146 PMC4343198

[B79] Hsu P. D. Scott D. A. Weinstein J. A. Ran F. A. Konermann S. Agarwala V.. (2013). DNA targeting specificity of RNA-guided Cas9 nucleases. Nat. Biotechnol. 31, 827–832. 10.1038/nbt.2647 23873081 PMC3969858

[B80] Hu J. Meyers R. M. Dong J. Panchakshari R. A. Alt F. W. Frock R. L. (2016). Detecting DNA double-stranded breaks in mammalian genomes by linear amplification-mediated high-throughput genome-wide translocation sequencing. Nat. Protoc. 11, 853–871. 10.1038/nprot.2016.043 27031497 PMC4895203

[B81] Hu J. H. Miller S. M. Geurts M. H. Tang W. Chen L. Sun N.. (2018). Evolved Cas9 variants with broad PAM compatibility and high DNA specificity. Nature 556, 57–63. 10.1038/nature26155 29512652 PMC5951633

[B82] Hu W. Kaminski R. Yang F. Zhang Y. Cosentino L. Li F.. (2014). RNA-directed gene editing specifically eradicates latent and prevents new HIV-1 infection. Proc. Natl. Acad. Sci. U.S.A. 111, 11461–11466. 10.1073/pnas.1405186111 25049410 PMC4128125

[B83] Huang J. Wang Y. Zhao J. (2018). CRISPR editing in biological and biomedical investigation. J. Cell. Physiol. 233, 3875–3891. 10.1002/jcp.26141 28786481

[B84] Iacovoni J. S. Caron P. Lassadi I. Nicolas E. Massip L. Trouche D.. (2010). High-resolution profiling of gammaH2AX around DNA double strand breaks in the mammalian genome. EMBO J. 29, 1446–1457. 10.1038/emboj.2010.38 20360682 PMC2868577

[B85] Iyer V. Shen B. Zhang W. Hodgkins A. Keane T. Huang X.. (2015). Off-target mutations are rare in Cas9-modified mice. Nat. Methods 12, 479–479. 10.1038/nmeth.3408 26020497

[B86] Jensen K. T. Floe L. Petersen T. S. Huang J. Xu F. Bolund L.. (2017). Chromatin accessibility and guide sequence secondary structure affect CRISPR-Cas9 gene editing efficiency. FEBS Lett. 591, 1892–1901. 10.1002/1873-3468.12707 28580607

[B87] Ji H. Jiang Z. Lu P. Ma L. Li C. Pan H.. (2016). Specific reactivation of latent HIV-1 by dCas9-SunTag-VP64-mediated guide RNA Targeting the HIV-1 promoter. Mol. Ther. 24, 508–521. 10.1038/mt.2016.7 26775808 PMC4786936

[B88] Jinek M. Chylinski K. Fonfara I. Hauer M. Doudna J. A. Charpentier E. (2012). A programmable dual-RNA-guided DNA endonuclease in adaptive bacterial immunity. Science 337, 816–821. 10.1126/science.1225829 22745249 PMC6286148

[B89] Jinek M. East A. Cheng A. Lin S. Ma E. Doudna J. (2013). RNA-programmed genome editing in human cells. Elife 2:e00471. 10.7554/eLife.00471.009 23386978 PMC3557905

[B90] Johansson H. Isaksson M. Sorqvist E. F. Roos F. Stenberg J. Sjoblom T.. (2011). Targeted resequencing of candidate genes using selector probes. Nucleic Acids Res. 39:e8. 10.1093/nar/gkq1005 21059679 PMC3025563

[B91] Kaminski R. Bella R. Yin C. Otte J. Ferrante P. Gendelman H. E.. (2016a). Excision of HIV-1 DNA by gene editing: a proof-of-concept *in vivo* study. Gene Ther. 23, 690–695. 10.1038/gt.2016.41 27194423 PMC4974122

[B92] Kaminski R. Chen Y. Fischer T. Tedaldi E. Napoli A. Zhang Y.. (2016b). Elimination of HIV-1 genomes from human T-lymphoid cells by CRISPR/Cas9 gene editing. Sci. Rep. 6:22555. 10.1038/srep28213 26939770 PMC4778041

[B93] Kaminski R. Chen Y. Salkind J. Bella R. Young W. B. Ferrante P.. (2016c). Negative feedback regulation of HIV-1 by gene editing strategy. Sci. Rep. 6:31527. 10.1038/srep31527 27528385 PMC4985742

[B94] Karimian A. Azizian K. Parsian H. Rafieian S. Shafiei-Irannejad V. Kheyrollah M.. (2019). CRISPR/Cas9 technology as a potent molecular tool for gene therapy. J. Cell. Physiol. 234, 12267–12277. 10.1002/jcp.27972 30697727 PMC13484547

[B95] Kaushik A. Yndart A. Atluri V. Tiwari S. Tomitaka A. Gupta P.. (2019). Magnetically guided non-invasive CRISPR-Cas9/gRNA delivery across blood-brain barrier to eradicate latent HIV-1 infection. Sci. Rep. 9:3928. 10.1038/s41598-019-40222-4 30850620 PMC6408460

[B96] Khair L. Baker R. E. Linehan E. K. Schrader C. E. Stavnezer J. (2015). Nbs1 ChIP-Seq identifies off-target DNA double-strand breaks induced by AID in activated splenic B cells. PLoS Genet. 11:e1005438. 10.1371/journal.pgen.1005438 26263206 PMC4532491

[B97] Kim D. Bae S. Park J. Kim E. Kim S. Yu H. R.. (2015). Digenome-seq: genome-wide profiling of CRISPR-Cas9 off-target effects in human cells. Nat. Methods 12, 237–243. 10.1038/nmeth.3284 25664545

[B98] Kim D. Kim J. S. (2018). DIG-seq: a genome-wide CRISPR off-target profiling method using chromatin DNA. Genome Res. 28, 1894–1900. 10.1101/gr.236620.118 30413470 PMC6280750

[B99] Kim D. Kim S. Kim S. Park J. Kim J. S. (2016). Genome-wide target specificities of CRISPR-Cas9 nucleases revealed by multiplex Digenome-seq. Genome Res. 26, 406–415. 10.1101/gr.199588.115 26786045 PMC4772022

[B100] Kim H. J. Lee H. J. Kim H. Cho S. W. Kim J. S. (2009). Targeted genome editing in human cells with zinc finger nucleases constructed via modular assembly. Genome Res. 19, 1279–1288. 10.1101/gr.089417.108 19470664 PMC2704428

[B101] Kim T. H. Ren B. (2006). Genome-wide analysis of protein-DNA interactions. Annu. Rev. Genomics Hum. Genet. 7, 81–102. 10.1146/annurev.genom.7.080505.115634 16722805

[B102] Klein I. A. Resch W. Jankovic M. Oliveira T. Yamane A. Nakahashi H.. (2011). Translocation-capture sequencing reveals the extent and nature of chromosomal rearrangements in B lymphocytes. Cell 147, 95–106. 10.1016/j.cell.2011.07.048 21962510 PMC3190307

[B103] Kleinstiver B. P. Pattanayak V. Prew M. S. Tsai S. Q. Nguyen N. T. Zheng Z.. (2016). High-fidelity CRISPR-Cas9 nucleases with no detectable genome-wide off-target effects. Nature 529, 490–495. 10.1038/nature16526 26735016 PMC4851738

[B104] Knight S. C. Xie L. Deng W. Guglielmi B. Witkowsky L. B. Bosanac L.. (2015). Dynamics of CRISPR-Cas9 genome interrogation in living cells. Science 350, 823–826. 10.1126/science.aac6572 26564855

[B105] Koo T. Lee J. Kim J. S. (2015). Measuring and reducing off-target activities of programmable nucleases including CRISPR-Cas9. Mol. Cells 38, 475–481. 10.14348/molcells.2015.0103 25985872 PMC4469905

[B106] Kosicki M. Tomberg K. Bradley A. (2018). Repair of double-strand breaks induced by CRISPR-Cas9 leads to large deletions and complex rearrangements. Nat. Biotechnol. 36, 765–771. 10.1038/nbt.4192 30010673 PMC6390938

[B107] Kunze C. Borner K. Kienle E. Orschmann T. Rusha E. Schneider M.. (2018). Synthetic AAV/CRISPR vectors for blocking HIV-1 expression in persistently infected astrocytes. Glia 66, 413–427. 10.1002/glia.23254 29119608

[B108] Kuscu C. Arslan S. Singh R. Thorpe J. Adli M. (2014). Genome-wide analysis reveals characteristics of off-target sites bound by the Cas9 endonuclease. Nat. Biotechnol. 32, 677–683. 10.1038/nbt.2916 24837660

[B109] Lazzarotto C. R. Malinin N. L. Li Y. Zhang R. Yang Y. Lee G.. (2020). CHANGE-seq reveals genetic and epigenetic effects on CRISPR-Cas9 genome-wide activity. Nat. Biotechnol. 38, 1317–1327. 10.1038/s41587-020-0555-7 32541958 PMC7652380

[B110] Lazzarotto C. R. Nguyen N. T. Tang X. Malagon-Lopez J. Guo J. A. Aryee M. J.. (2018). Defining CRISPR-Cas9 genome-wide nuclease activities with CIRCLE-seq. Nat. Protoc. 13, 2615–2642. 10.1038/s41596-018-0055-0 30341435 PMC6512799

[B111] Lebbink R. J. de Jong D. C. Wolters F. Kruse E. M. van Ham P. M. Wiertz E. J.. (2017). A combinational CRISPR/Cas9 gene-editing approach can halt HIV replication and prevent viral escape. Sci. Rep. 7:41968. 10.1038/srep41968 28176813 PMC5296774

[B112] Lee J. K. Jeong E. Lee J. Jung M. Shin E. Kim Y. H.. (2018). Directed evolution of CRISPR-Cas9 to increase its specificity. Nat. Commun. 9:3048. 10.1038/s41467-018-05477-x 30082838 PMC6078992

[B113] Lensing S. V. Marsico G. Hansel-Hertsch R. Lam E. Y. Tannahill D. Balasubramanian S. (2016). DSBCapture: *in situ* capture and sequencing of DNA breaks. Nat. Methods 13, 855–857. 10.1038/nmeth.3960 27525976 PMC5045719

[B114] Li T. Zhu L. Xiao B. Gong Z. Liao Q. Guo J. (2019). CRISPR-Cpf1-mediated genome editing and gene regulation in human cells. Biotechnol. Adv. 37, 21–27. 10.1016/j.biotechadv.2018.10.013 30399413

[B115] Liang X. Potter J. Kumar S. Ravinder N. Chesnut J. D. (2017). Enhanced CRISPR/Cas9-mediated precise genome editing by improved design and delivery of gRNA, Cas9 nuclease, and donor DNA. J. Biotechnol. 241, 136–146. 10.1016/j.jbiotec.2016.11.011 27845164

[B116] Liang X. Potter J. Kumar S. Zou Y. Quintanilla R. Sridharan M.. (2015). Rapid and highly efficient mammalian cell engineering via Cas9 protein transfection. J. Biotechnol. 208, 44–53. 10.1016/j.jbiotec.2015.04.024 26003884

[B117] Liao H. K. Gu Y. Diaz A. Marlett J. Takahashi Y. Li M.. (2015). Use of the CRISPR/Cas9 system as an intracellular defense against HIV-1 infection in human cells. Nat. Commun. 6:6413. 10.1038/ncomms7413 25752527

[B118] Liao S. Tammaro M. Yan H. (2016). The structure of ends determines the pathway choice and Mre11 nuclease dependency of DNA double-strand break repair. Nucleic Acids Res. 44, 5689–5701. 10.1093/nar/gkw274 27084932 PMC4937313

[B119] Limsirichai P. Gaj T. Schaffer D. V. (2016). CRISPR-mediated activation of latent HIV-1 expression. Mol. Ther. 24, 499–507. 10.1038/mt.2015.213 26607397 PMC4786916

[B120] Lin Y. Cradick T. J. Brown M. T. Deshmukh H. Ranjan P. Sarode N.. (2014). CRISPR/Cas9 systems have off-target activity with insertions or deletions between target DNA and guide RNA sequences. Nucleic Acids Res. 42, 7473–7485. 10.1093/nar/gku402 24838573 PMC4066799

[B121] Link R. W. Nonnemacher M. R. Wigdahl B. Dampier W. (2018). Prediction of human immunodeficiency virus type 1 subtype-specific off-target effects arising from CRISPR-Cas9 gene editing therapy. CRISPR J. 1, 294–302. 10.1089/crispr.2018.0020 31021222 PMC6553478

[B122] Liu H. Wei Z. Dominguez A. Li Y. Wang X. Qi L. S. (2015). CRISPR-ERA: a comprehensive design tool for CRISPR-mediated gene editing, repression and activation. Bioinformatics 31, 3676–3678. 10.1093/bioinformatics/btv423 26209430 PMC4757951

[B123] Luo G. Yao M. S. Bender C. F. Mills M. Bladl A. R. Bradley A.. (1999). Disruption of mRad50 causes embryonic stem cell lethality, abnormal embryonic development, and sensitivity to ionizing radiation. Proc. Natl. Acad. Sci. U.S.A. 96, 7376–7381. 10.1073/pnas.96.13.7376 10377422 PMC22093

[B124] Madabhushi R. Gao F. Pfenning A. R. Pan L. Yamakawa S. Seo J.. (2015). Activity-induced DNA breaks govern the expression of neuronal early-response genes. Cell 161, 1592–1605. 10.1016/j.cell.2015.05.032 26052046 PMC4886855

[B125] Mali P. Aach J. Stranges P. B. Esvelt K. M. Moosburner M. Kosuri S.. (2013). CAS9 transcriptional activators for target specificity screening and paired nickases for cooperative genome engineering. Nat. Biotechnol. 31, 833–838. 10.1038/nbt.2675 23907171 PMC3818127

[B126] Mancuso P. Chen C. Kaminski R. Gordon J. Liao S. Robinson J. A.. (2020). CRISPR based editing of SIV proviral DNA in ART treated non-human primates. Nat. Commun. 11:6065. 10.1038/s41467-020-19821-7 33247091 PMC7695718

[B127] Manghwar H. Li B. Ding X. Hussain A. Lindsey K. Zhang X.. (2020). CRISPR/Cas systems in genome editing: methodologies and tools for sgRNA design, off-target evaluation, and strategies to mitigate off-target effects. Adv. Sci. 7:1902312. 10.1002/advs.201902312 32195078 PMC7080517

[B128] Mefferd A. L. Bogerd H. P. Irwan I. D. Cullen B. R. (2018). Insights into the mechanisms underlying the inactivation of HIV-1 proviruses by CRISPR/Cas. Virology 520, 116–126. 10.1016/j.virol.2018.05.016 29857168 PMC6100742

[B129] Meier J. A. Zhang F. Sanjana N. E. (2017). GUIDES: sgRNA design for loss-of-function screens. Nat. Methods 14, 831–832. 10.1038/nmeth.4423 28858339 PMC5870754

[B130] Meynert A. M. Ansari M. FitzPatrick D. R. Taylor M. S. (2014). Variant detection sensitivity and biases in whole genome and exome sequencing. BMC Bioinformatics 15:247. 10.1186/1471-2105-15-247 25038816 PMC4122774

[B131] Miller J. C. Holmes M. C. Wang J. Guschin D. Y. Lee Y. L. Rupniewski I.. (2007). An improved zinc-finger nuclease architecture for highly specific genome editing. Nat. Biotechnol. 25, 778–785. 10.1038/nbt1319 17603475

[B132] Montague T. G. Cruz J. M. Gagnon J. A. Church G. M. Valen E. (2014). CHOPCHOP: a CRISPR/Cas9 and TALEN web tool for genome editing. Nucleic Acids Res. 42, W401–407. 10.1093/nar/gku410 24861617 PMC4086086

[B133] Moreno-Herrero F. de Jager M. Dekker N. H. Kanaar R. Wyman C. Dekker C. (2005). Mesoscale conformational changes in the DNA-repair complex Rad50/Mre11/Nbs1 upon binding DNA. Nature 437, 440–443. 10.1038/nature03927 16163361

[B134] Murray A. J. Kwon K. J. Farber D. L. Siliciano R. F. (2016). The latent reservoir for HIV-1: how immunologic memory and clonal expansion contribute to HIV-1 persistence. J. Immunol. 197, 407–417. 10.4049/jimmunol.1600343 27382129 PMC4936486

[B135] Mussolino C. Morbitzer R. Lutge F. Dannemann N. Lahaye T. Cathomen T. (2011). A novel TALE nuclease scaffold enables high genome editing activity in combination with low toxicity. Nucleic Acids Res. 39, 9283–9293. 10.1093/nar/gkr597 21813459 PMC3241638

[B136] Neiman M. Sundling S. Gronberg H. Hall P. Czene K. Lindberg J.. (2012). Library preparation and multiplex capture for massive parallel sequencing applications made efficient and easy. PLoS ONE 7:e48616. 10.1371/journal.pone.0048616 23139805 PMC3489721

[B137] Nobles C. L. Reddy S. Salas-McKee J. Liu X. June C. H. Melenhorst J. J.. (2019). iGUIDE: an improved pipeline for analyzing CRISPR cleavage specificity. Genome Biol. 20:14. 10.1186/s13059-019-1625-3 30654827 PMC6337799

[B138] O'Geen H. Henry I. M. Bhakta M. S. Meckler J. F. Segal D. J. (2015). A genome-wide analysis of Cas9 binding specificity using ChIP-seq and targeted sequence capture. Nucleic Acids Res. 43, 3389–3404. 10.1093/nar/gkv137 25712100 PMC4381059

[B139] Oh J. Symington L. S. (2018). Role of the Mre11 complex in preserving genome integrity. Genes 9. 10.3390/genes9120589 30501098 PMC6315862

[B140] Ophinni Y. Inoue M. Kotaki T. Kameoka M. (2018). CRISPR/Cas9 system targeting regulatory genes of HIV-1 inhibits viral replication in infected T-cell cultures. Sci. Rep. 8:7784. 10.1038/s41598-018-26190-1 29773895 PMC5958087

[B141] Panfil A. R. London J. A. Green P. L. Yoder K. E. (2018). CRISPR/Cas9 genome editing to disable the latent HIV-1 provirus. Front. Microbiol. 9:3107. 10.3389/fmicb.2018.03107 30619186 PMC6302043

[B142] Paruzynski A. Arens A. Gabriel R. Bartholomae C. C. Scholz S. Wang W.. (2010). Genome-wide high-throughput integrome analyses by nrLAM-PCR and next-generation sequencing. Nat. Protoc. 5, 1379–1395. 10.1038/nprot.2010.87 20671722

[B143] Pattanayak V. Lin S. Guilinger J. P. Ma E. Doudna J. A. Liu D. R. (2013). High-throughput profiling of off-target DNA cleavage reveals RNA-programmed Cas9 nuclease specificity. Nat. Biotechnol. 31, 839–843. 10.1038/nbt.2673 23934178 PMC3782611

[B144] Pattanayak V. Ramirez C. L. Joung J. K. Liu D. R. (2011). Revealing off-target cleavage specificities of zinc-finger nucleases by *in vitro* selection. Nat. Methods 8, 765–770. 10.1038/nmeth.1670 21822273 PMC3164905

[B145] Picelli S. Bjorklund A. K. Reinius B. Sagasser S. Winberg G. Sandberg R. (2014). Tn5 transposase and tagmentation procedures for massively scaled sequencing projects. Genome Res. 24, 2033–2040. 10.1101/gr.177881.114 25079858 PMC4248319

[B146] Pollack R. A. Jones R. B. Pertea M. Bruner K. M. Martin A. R. Thomas A. S.. (2017). Defective HIV-1 proviruses are expressed and can be recognized by cytotoxic T lymphocytes, which shape the proviral landscape. Cell Host Microbe 21, 494–506 e494. 10.1016/j.chom.2017.03.008 28407485 PMC5433942

[B147] Qi J. Ding C. Jiang X. Gao Y. (2020). Advances in developing CAR T-cell therapy for HIV cure. Front. Immunol. 11:361. 10.3389/fimmu.2020.00361 32210965 PMC7076163

[B148] Ramiro A. R. Jankovic M. Callen E. Difilippantonio S. Chen H. T. McBride K. M.. (2006). Role of genomic instability and p53 in AID-induced c-myc-Igh translocations. Nature 440, 105–109. 10.1038/nature04495 16400328 PMC4601098

[B149] Rodriguez R. Miller K. M. Forment J. V. Bradshaw C. R. Nikan M. Britton S.. (2012). Small-molecule-induced DNA damage identifies alternative DNA structures in human genes. Nat. Chem. Biol. 8, 301–310. 10.1038/nchembio.780 22306580 PMC3433707

[B150] Roychoudhury P. De Silva Feelixge H. Reeves D. Mayer B. T. Stone D. Schiffer J. T.. (2018). Viral diversity is an obligate consideration in CRISPR/Cas9 designs for targeting the HIV reservoir. BMC Biol. 16:75. 10.1186/s12915-018-0544-1 29996827 PMC6040082

[B151] Saayman S. M. Lazar D. C. Scott T. A. Hart J. R. Takahashi M. Burnett J. C.. (2016). Potent and targeted activation of latent HIV-1 using the CRISPR/dCas9 activator complex. Mol. Ther. 24, 488–498. 10.1038/mt.2015.202 26581162 PMC4786915

[B152] Schmid-Burgk J. L. Gao L. Li D. Gardner Z. Strecker J. Lash B.. (2020). Highly parallel profiling of Cas9 variant specificity. Mol. Cell. 78, 794–800. 10.1016/j.molcel.2020.02.023 32187529 PMC7370240

[B153] Schmidt M. Schwarzwaelder K. Bartholomae C. Zaoui K. Ball C. Pilz I.. (2007). High-resolution insertion-site analysis by linear amplification-mediated PCR (LAM-PCR). Nat. Methods 4, 1051–1057. 10.1038/nmeth1103 18049469

[B154] Shapiro J. Iancu O. Jacobi A. M. McNeill M. S. Turk R. Rettig G. R.. (2020). Increasing CRISPR efficiency and measuring its specificity in HSPCs using a clinically relevant system. Mol. Ther. Methods Clin. Dev. 17, 1097–1107. 10.1016/j.omtm.2020.04.027 32478125 PMC7251314

[B155] Shibata A. Moiani D. Arvai A. S. Perry J. Harding S. M. Genois M. M.. (2014). DNA double-strand break repair pathway choice is directed by distinct MRE11 nuclease activities. Mol. Cell 53, 7–18. 10.1016/j.molcel.2013.11.003 24316220 PMC3909494

[B156] Siliciano R. F. Greene W. C. (2011). HIV latency. Cold Spring Harb. Perspect. Med. 1:a007096. 10.1101/cshperspect.a007096 22229121 PMC3234450

[B157] Slaymaker I. M. Gao L. Zetsche B. Scott D. A. Yan W. X. Zhang F. (2016). Rationally engineered Cas9 nucleases with improved specificity. Science 351, 84–88. 10.1126/science.aad5227 26628643 PMC4714946

[B158] Smith C. Gore A. Yan W. Abalde-Atristain L. Li Z. He C.. (2014). Whole-genome sequencing analysis reveals high specificity of CRISPR/Cas9 and TALEN-based genome editing in human iPSCs. Cell Stem Cell 15, 12–13. 10.1016/j.stem.2014.06.011 24996165 PMC4338993

[B159] Spehalski E. Capper K. M. Smith C. J. Morgan M. J. Dinkelmann M. Buis J.. (2017). MRE11 promotes tumorigenesis by facilitating resistance to oncogene-induced replication stress. Cancer Res. 77, 5327–5338. 10.1158/0008-5472.CAN-17-1355 28819025 PMC5831255

[B160] Staszewski O. Baker R. E. Ucher A. J. Martier R. Stavnezer J. Guikema J. E. (2011). Activation-induced cytidine deaminase induces reproducible DNA breaks at many non-Ig Loci in activated B cells. Mol. Cell 41, 232–242. 10.1016/j.molcel.2011.01.007 21255732 PMC3044441

[B161] Su H. Sravanam S. Gorantla S. Kaminski R. Khalili K. Poluektova L.. (2020). Amplification of replication competent HIV-1 by adoptive transfer of human cells from infected humanized mice. Front. Cell. Infect. Microbiol. 10:38. 10.3389/fcimb.2020.00038 32117811 PMC7026001

[B162] Sullivan N. T. Dampier W. Chung C. H. Allen A. G. Atkins A. Pirrone V.. (2019). Novel gRNA design pipeline to develop broad-spectrum CRISPR/Cas9 gRNAs for safe targeting of the HIV-1 quasispecies in patients. Sci. Rep. 9:17088. 10.1038/s41598-019-52353-9 31745112 PMC6864089

[B163] Suzuki K. Yu C. Qu J. Li M. Yao X. Yuan T.. (2014). Targeted gene correction minimally impacts whole-genome mutational load in human-disease-specific induced pluripotent stem cell clones. Cell Stem Cell 15, 31–36. 10.1016/j.stem.2014.06.016 24996168 PMC4144407

[B164] Syed A. Tainer J. A. (2018). The MRE11-RAD50-NBS1 complex conducts the orchestration of damage signaling and outcomes to stress in DNA replication and repair. Annu. Rev. Biochem. 87, 263–294. 10.1146/annurev-biochem-062917-012415 29709199 PMC6076887

[B165] Szczepek M. Brondani V. Buchel J. Serrano L. Segal D. J. Cathomen T. (2007). Structure-based redesign of the dimerization interface reduces the toxicity of zinc-finger nucleases. Nat. Biotechnol. 25, 786–793. 10.1038/nbt1317 17603476

[B166] Szilard R. K. Jacques P. E. Laramee L. Cheng B. Galicia S. Bataille A. R.. (2010). Systematic identification of fragile sites via genome-wide location analysis of gamma-H2AX. Nat. Struct. Mol. Biol. 17, 299–305. 10.1038/nsmb.1754 20139982 PMC3081315

[B167] Tsai S. Q. Joung J. K. (2016). Defining and improving the genome-wide specificities of CRISPR-Cas9 nucleases. Nat. Rev. Genet. 17, 300–312. 10.1038/nrg.2016.28 27087594 PMC7225572

[B168] Tsai S. Q. Nguyen N. T. Malagon-Lopez J. Topkar V. V. Aryee M. J. Joung J. K. (2017). CIRCLE-seq: a highly sensitive *in vitro* screen for genome-wide CRISPR-Cas9 nuclease off-targets. Nat. Methods 14, 607–614. 10.1038/nmeth.4278 28459458 PMC5924695

[B169] Tsai S. Q. Zheng Z. Nguyen N. T. Liebers M. Topkar V. V. Thapar V.. (2015). GUIDE-seq enables genome-wide profiling of off-target cleavage by CRISPR-Cas nucleases. Nat. Biotechnol. 33, 187–197. 10.1038/nbt.3117 25513782 PMC4320685

[B170] Tycko J. Myer V. E. Hsu P. D. (2016). Methods for optimizing CRISPR-Cas9 genome editing specificity. Mol. Cell 63, 355–370. 10.1016/j.molcel.2016.07.004 27494557 PMC4976696

[B171] Ueda S. Ebina H. Kanemura Y. Misawa N. Koyanagi Y. (2016). Anti-HIV-1 potency of the CRISPR/Cas9 system insufficient to fully inhibit viral replication. Microbiol. Immunol. 60, 483–496. 10.1111/1348-0421.12395 27278725

[B172] Vakulskas C. A. Dever D. P. Rettig G. R. Turk R. Jacobi A. M. Collingwood M. A.. (2018). A high-fidelity Cas9 mutant delivered as a ribonucleoprotein complex enables efficient gene editing in human hematopoietic stem and progenitor cells. Nat. Med. 24, 1216–1224. 10.1038/s41591-018-0137-0 30082871 PMC6107069

[B173] van den Bosch M. Bree R. T. Lowndes N. F. (2003). The MRN complex: coordinating and mediating the response to broken chromosomes. EMBO Rep. 4, 844–849. 10.1038/sj.embor.embor925 12949583 PMC1326362

[B174] Varley K. E. Mitra R. D. (2008). Nested Patch PCR enables highly multiplexed mutation discovery in candidate genes. Genome Res. 18, 1844–1850. 10.1101/gr.078204.108 18849522 PMC2577855

[B175] Veres A. Gosis B. S. Ding Q. Collins R. Ragavendran A. Brand H.. (2014). Low incidence of off-target mutations in individual CRISPR-Cas9 and TALEN targeted human stem cell clones detected by whole-genome sequencing. Cell Stem Cell 15, 27–30. 10.1016/j.stem.2014.04.020 24996167 PMC4082799

[B176] Vouillot L. Thelie A. Pollet N. (2015). Comparison of T7E1 and surveyor mismatch cleavage assays to detect mutations triggered by engineered nucleases. G3 5, 407–415. 10.1534/g3.114.015834 25566793 PMC4349094

[B177] Wang G. Zhao N. Berkhout B. Das A. T. (2016a). A combinatorial CRISPR-Cas9 attack on HIV-1 DNA extinguishes all infectious provirus in infected T cell cultures. Cell. Rep. 17, 2819–2826. 10.1016/j.celrep.2016.11.057 27974196

[B178] Wang G. Zhao N. Berkhout B. Das A. T. (2016b). CRISPR-Cas9 can inhibit HIV-1 replication but NHEJ repair facilitates virus escape. Mol. Ther. 24, 522–526. 10.1038/mt.2016.24 26796669 PMC4786927

[B179] Wang J. Zhang X. Cheng L. Luo Y. (2020). An overview and metanalysis of machine and deep learning-based CRISPR gRNA design tools. RNA Biol. 17, 13–22. 10.1080/15476286.2019.1669406 31533522 PMC6948960

[B180] Wang X. Wang Y. Wu X. Wang J. Wang Y. Qiu Z.. (2015). Unbiased detection of off-target cleavage by CRISPR-Cas9 and TALENs using integrase-defective lentiviral vectors. Nat. Biotechnol. 33, 175–178. 10.1038/nbt.3127 25599175

[B181] Wang Z. Wang W. Cui Y. C. Pan Q. Zhu W. Gendron P.. (2018). HIV-1 employs multiple mechanisms to resist Cas9/single guide RNA targeting the viral primer binding site. J. Virol. 92. 10.1128/JVI.01135-18 30068653 PMC6158435

[B182] Wienert B. Wyman S. K. Richardson C. D. Yeh C. D. Akcakaya P. Porritt M. J.. (2019). Unbiased detection of CRISPR off-targets *in vivo* using DISCOVER-Seq. Science 364, 286–289. 10.1101/469635 31000663 PMC6589096

[B183] Wienert B. Wyman S. K. Yeh C. D. Conklin B. R. Corn J. E. (2020). CRISPR off-target detection with DISCOVER-seq. Nat. Protoc. 15, 1775–1799. 10.1038/s41596-020-0309-5 32313254 PMC7305837

[B184] Wu X. Scott D. A. Kriz A. J. Chiu A. C. Hsu P. D. Dadon D. B.. (2014). Genome-wide binding of the CRISPR endonuclease Cas9 in mammalian cells. Nat. Biotechnol. 32, 670–676. 10.1038/nbt.2889 24752079 PMC4145672

[B185] Xu L. Yang H. Gao Y. Chen Z. Xie L. Liu Y.. (2017). CRISPR/Cas9-mediated CCR5 ablation in human hematopoietic stem/progenitor cells confers HIV-1 resistance *in vivo*. Mol. Ther. 25, 1782–1789. 10.1016/j.ymthe.2017.04.027 28527722 PMC5542791

[B186] Yamane A. Robbiani D. F. Resch W. Bothmer A. Nakahashi H. Oliveira T.. (2013). RPA accumulation during class switch recombination represents 5'-3′ DNA-end resection during the S-G2/M phase of the cell cycle. Cell. Rep. 3, 138–147. 10.1016/j.celrep.2012.12.006 23291097 PMC3563767

[B187] Yan W. X. Mirzazadeh R. Garnerone S. Scott D. Schneider M. W. Kallas T.. (2017). BLISS is a versatile and quantitative method for genome-wide profiling of DNA double-strand breaks. Nat. Commun. 8:15058. 10.1038/ncomms15058 28497783 PMC5437291

[B188] Yang H. Wang H. Shivalila C. S. Cheng A. W. Shi L. Jaenisch R. (2013). One-step generation of mice carrying reporter and conditional alleles by CRISPR/Cas-mediated genome engineering. Cell 154, 1370–1379. 10.1016/j.cell.2013.08.022 23992847 PMC3961003

[B189] Yang Z. Steentoft C. Hauge C. Hansen L. Thomsen A. L. Niola F.. (2015). Fast and sensitive detection of indels induced by precise gene targeting. Nucleic Acids Res. 43:e59. 10.1093/nar/gkv126 25753669 PMC4482057

[B190] Yin C. Zhang T. Li F. Yang F. Putatunda R. Young W. B.. (2016). Functional screening of guide RNAs targeting the regulatory and structural HIV-1 viral genome for a cure of AIDS. AIDS 30, 1163–1174. 10.1097/QAD.0000000000001079 26990633 PMC4851589

[B191] Yoder K. E. Bundschuh R. (2016). Host double strand break repair generates HIV-1 strains resistant to CRISPR/Cas9. Sci. Rep. 6:29530. 10.1038/srep29530 27404981 PMC4941621

[B192] Yousem S. A. Dacic S. Nikiforov Y. E. Nikiforova M. (2013). Pulmonary Langerhans cell histiocytosis: profiling of multifocal tumors using next-generation sequencing identifies concordant occurrence of BRAF V600E mutations. Chest 143, 1679–1684. 10.1378/chest.12-1917 23287985

[B193] Zeng J. Wu Y. Ren C. Bonanno J. Shen A. H. Shea D.. (2020). Therapeutic base editing of human hematopoietic stem cells. Nat. Med. 26, 535–541. 10.1038/s41591-020-0790-y 32284612 PMC7869435

[B194] Zhang D. Hurst T. Duan D. Chen S. J. (2019). Unified energetics analysis unravels SpCas9 cleavage activity for optimal gRNA design. Proc. Natl. Acad. Sci. U.S.A. 116, 8693–8698. 10.1073/pnas.1820523116 30988204 PMC6500161

[B195] Zhang H. Zhang J. Wei P. Zhang B. Gou F. Feng Z.. (2014). The CRISPR/Cas9 system produces specific and homozygous targeted gene editing in rice in one generation. Plant Biotechnol. J. 12, 797–807. 10.1111/pbi.12200 24854982

[B196] Zhao N. Wang G. Das A. T. Berkhout B. (2017). Combinatorial CRISPR-Cas9 and RNA interference attack on HIV-1 DNA and RNA can lead to cross-resistance. Antimicrob. Agents Chemother. 61. 10.1128/AAC.01486-17 28893790 PMC5700367

[B197] Zheng Z. Advani A. Melefors O. Glavas S. Nordstrom H. Ye W.. (2010). Titration-free massively parallel pyrosequencing using trace amounts of starting material. Nucleic Acids Res. 38:e137. 10.1093/nar/gkq332 20435675 PMC2910068

[B198] Zheng Z. Advani A. Melefors O. Glavas S. Nordstrom H. Ye W.. (2011). Titration-free 454 sequencing using Y adapters. Nat. Protoc. 6, 1367–1376. 10.1038/nprot.2011.369 21886102

[B199] Zheng Z. Liebers M. Zhelyazkova B. Cao Y. Panditi D. Lynch K. D.. (2014). Anchored multiplex PCR for targeted next-generation sequencing. Nat. Med. 20, 1479–1484. 10.1038/nm.3729 25384085

[B200] Zhou Z. X. Zhang M. J. Peng X. Takayama Y. Xu X. Y. Huang L. Z.. (2013). Mapping genomic hotspots of DNA damage by a single-strand-DNA-compatible and strand-specific ChIP-seq method. Genome Res. 23, 705–715. 10.1101/gr.146357.112 23249883 PMC3613587

[B201] Zhu J. Petersen S. Tessarollo L. Nussenzweig A. (2001). Targeted disruption of the Nijmegen breakage syndrome gene NBS1 leads to early embryonic lethality in mice. Curr. Biol. 11, 105–109. 10.1016/S0960-9822(01)00019-7 11231126

[B202] Zhu W. Lei R. Le Duff Y. Li J. Guo F. Wainberg M. A.. (2015). The CRISPR/Cas9 system inactivates latent HIV-1 proviral DNA. Retrovirology 12:22. 10.1186/s12977-015-0150-z 25808449 PMC4359768

[B203] Zuo E. Sun Y. Wei W. Yuan T. Ying W. Sun H.. (2019). Cytosine base editor generates substantial off-target single-nucleotide variants in mouse embryos. Science 364, 289–292. 10.1126/science.aav9973 30819928 PMC7301308

